# Artificial disulfide-rich peptide scaffolds with precisely defined disulfide patterns and a minimized number of isomers†
†Electronic supplementary information (ESI) available: Experiments on the synthesis and characterization of peptides, the oxidative folding of peptides, and the tryptic digestion LC-MS analysis of disulfide connectivity. See DOI: 10.1039/c6sc05710a



**DOI:** 10.1039/c6sc05710a

**Published:** 2017-02-17

**Authors:** Yiwu Zheng, Zhuoru Li, Jing Ren, Weidong Liu, Yaqi Wu, Yibing Zhao, Chuanliu Wu

**Affiliations:** a The MOE Key Laboratory of Spectrochemical Analysis and Instrumentation , State Key Laboratory of Physical Chemistry of Solid Surfaces , Collaborative Innovation Center of Chemistry for Energy Materials , Department of Chemistry , College of Chemistry and Chemical Engineering , Xiamen University , Xiamen , 361005 , P.R. China . Email: chlwu@xmu.edu.cn

## Abstract

Disulfide-rich peptides are emerging as potential templates for drug design applications. However, the synthesis and reengineering of disulfide-rich peptides are challenging, owing to the complexity of the oxidative folding process involving a number of diverse isomeric structures. Novel disulfide-rich peptide scaffolds that are not besieged by their disulfide isomers are still greatly desired. In this work, we report the design and synthesis of a novel class of artificial disulfide-rich peptide scaffolds with precisely defined disulfide patterns and a minimized number of isomers. In theory, natural peptides with three disulfide bonds have 15 possible isomers. By rationally engineering the thiol-framework of a peptide containing six cysteines with penicillamines and a dithiol amino acid, we demonstrated, for the first time, that the total number of isomers formed after oxidative folding can be decreased to a minimum of two (*i.e.*, from 15 to 2). As fewer isomeric folds are involved in the oxidative folding, the pathway of the folding becomes more concise and the yield of the artificial scaffolds is substantially increased compared to that of its six-cysteine-containing analogue, which makes the artificial disulfide-rich scaffolds (with only 2 predefined isomeric folds) extremely promising for being exploited as structurally complex templates for the design of peptide therapeutics and ligands.

## Abstract 2

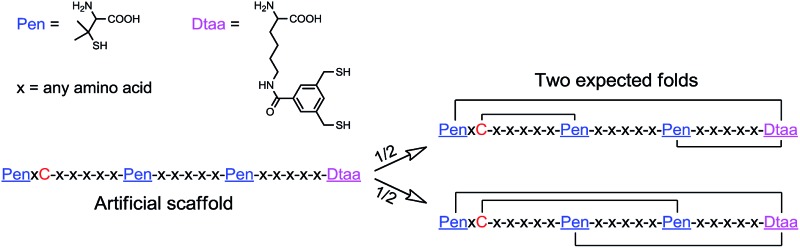
We report the design and synthesis of artificial disulfide-rich peptide scaffolds with precisely defined disulfide patterns and a minimized number of isomers.

## Introduction

The discovery and creation of novel organic structures or scaffolds has been a propeller driving the development of small-molecule drugs.^1^ The development of peptide-based drugs would also benefit from the discovery and synthesis of natural peptide scaffolds.^2^ Indeed, the huge diversity of disulfide-rich peptides, including antimicrobial defensins, plant-derived cyclotides, and conotoxins from the venom of predatory marine snails, holds great promise of being exploited for the development of novel therapeutics for diverse human diseases.^2a–e,3^ While short peptides are usually structurally ill-defined and extremely susceptible to enzymatic hydrolysis,^4^ these privileged peptides containing multiple disulfide bonds can significantly reduce conformational flexibility and fix the structures of peptides into their bioactive states, therefore displaying an improved binding efficiency and specificity, and can be more tolerant to proteolysis compared to linear peptides.^2e,3a,5^ However, the synthesis and reengineering of disulfide-rich peptides have been considered to be an overt challenge, mainly owing to the complexity of oxidative folding processes which amplifies rapidly as the number of possible isomers with different disulfide patterns increases.^6^

Regioselective approaches involving orthogonal protecting groups, stepwise deprotections, and oxidations are most often exploited to ensure the synthesis of the desired isomers,^7^ which is usually sophisticated and laborious; moreover, the desired isomer might evolve towards other disulfide isomers due to isomerization of the disulfide bonds under thermodynamic control in the presence of thiols in biological fluids.^8^ Alternative strategies exploited the orthogonal or preferential pairing between deprotected thiols (cysteine (Cys), penicillamine (Pen) or synthetic thiols) or the diselenide formation between selenocysteine residues to direct the oxidative folding of the peptides.^8,9^ Though these strategies usually afford the desired isomers as the major folding products, the undesired tendency of forming other disulfide isomers can still complicate the folding processes and trigger disulfide isomerizations, particularly when the disulfide-rich peptides are used as structural scaffolds for drug design applications, because in this circumstance, their primary sequences are subject to extensive manipulation. Accordingly, novel disulfide-rich peptide scaffolds that are not besieged by their disulfide isomers, and thus are more tolerant to sequence manipulation than conventional natural peptide scaffolds, are still greatly desired.

**Fig. 1:**
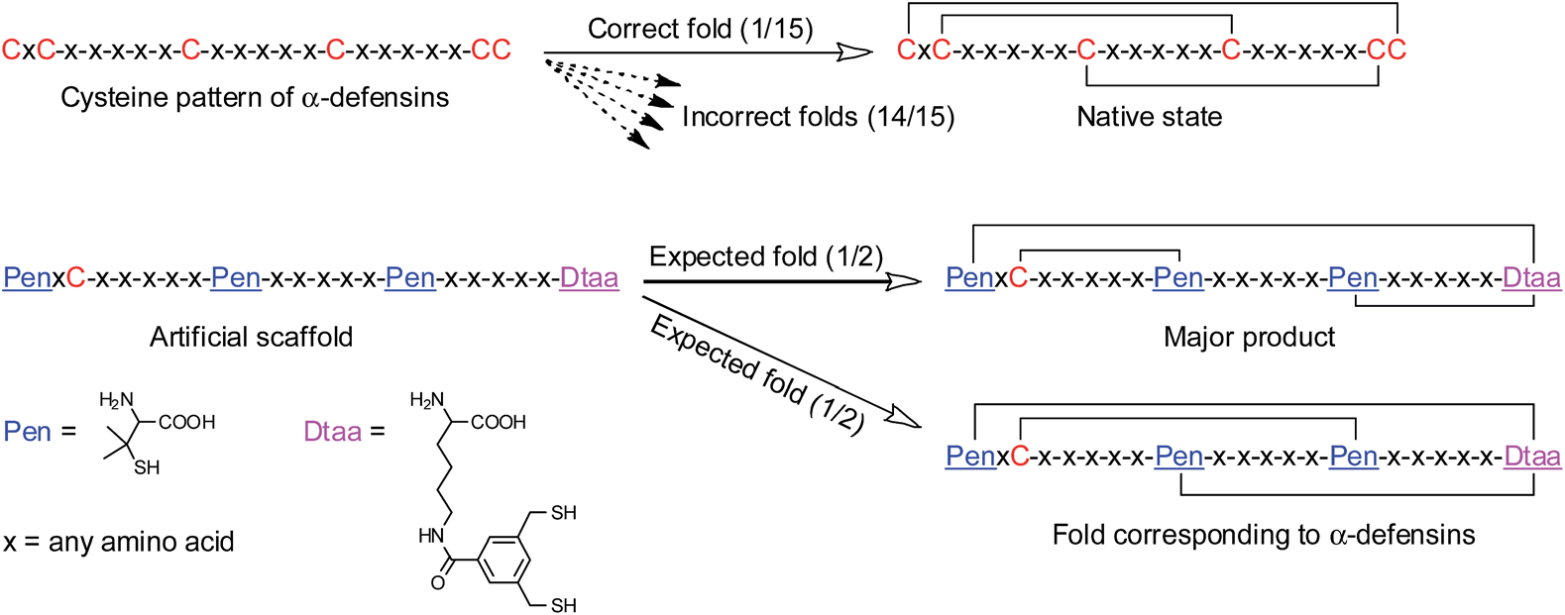
Illustration of the oxidative folding of peptides containing six cysteine residues (15 total folds, into which only one corresponds to the native fold); the artificial peptide scaffold designed in this work, which contains the unnatural amino acids Pen and Dtaa; only two expected folds formed after the oxidative folding of the peptides, and one of the two corresponds to the disulfide connectivity of human α-defensin 5 (but of less abundance). -x-x-x-x-x- denotes a peptide segment containing any number of natural amino acids of any type, except cysteine residues.

Here, we report the design and synthesis of artificial disulfide-rich peptide scaffolds with precisely defined disulfide connectivity and a minimized number of isomers. The artificial scaffolds have three disulfide bonds, which can spontaneously pair with a high degree of accuracy in redox buffers. In theory, natural peptides with three disulfide bonds can form 15 possible isomers corresponding to different disulfide connectivities. We strategically transformed a specific cysteine framework, which is naturally recruited for the production of human α-defensins (*i.e.*, CXC–C–C–CC),^2a,2b,10^ into several artificial thiol-frameworks containing three Pen residues and a synthetic dithiol amino acid (Dtaa) (Fig. 1). The oxidative folding of the peptides with unique C/Pen/Dtaa-frameworks leads to the formation of structurally pre-defined disulfide-rich peptide scaffolds, a process that is exclusively due to the directed pairing of the disulfide bonds, but not the sequence-specific prefolding. These artificial peptide scaffolds should be much more tolerant to sequence manipulation and, in principle, can be better at avoiding the problematic isomerization of disulfide bonds compared to natural ones.

## Results and discussion

**Fig. 2:**
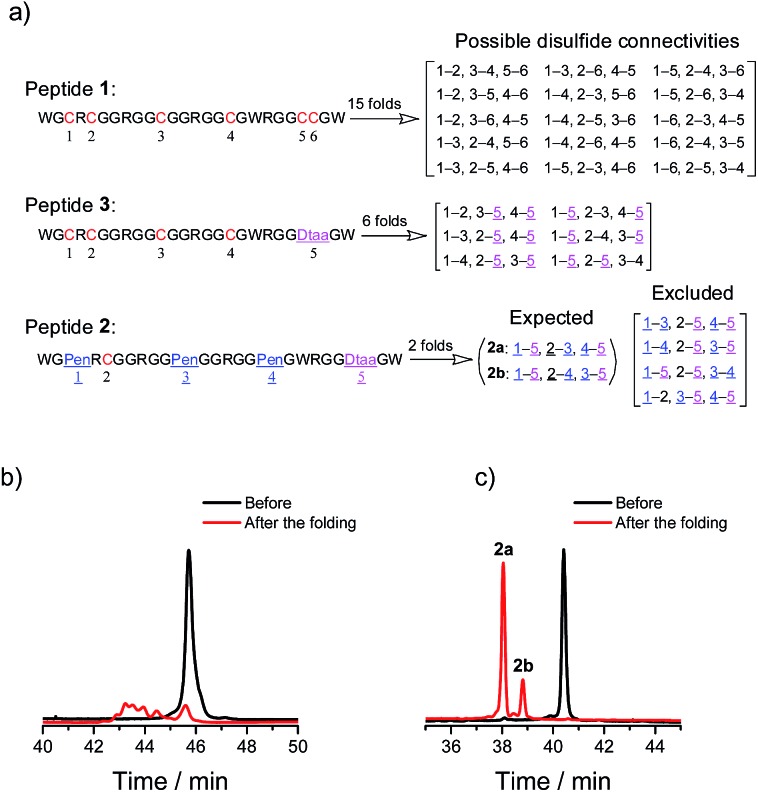
(a) Possible or expected isomers that might form after oxidative folding. (b) Chromatograms showing the oxidation of **1** (10 µM) in 0.2 mM GSSG and 50 vol% DMSO/100 mM phosphate buffer (pH 7.4) (black line: before the oxidation; red line: 2 h after oxidative folding). (c) Chromatograms showing two expected products formed after the oxidation of **2** (10 µM) in 0.2 mM GSSG and 50 vol% DMSO/100 mM phosphate buffer (pH 7.4) (black line: before the oxidation; red line: 2 h after the folding); the disulfide connectivity of **2a** and **2b**: 1–5, 2–3, 4–5 and 1–5, 2–4, 3–5, respectively (determined using tryptic digestion LC-MS).

A model glycine residue-rich peptide (**1**) patterned with the CXC–C–C–CC framework was first designed and synthesized, in which non-glycine amino acids were placed for the later analysis of cysteine pairing in the oxidized products using tryptic digestion liquid chromatography-mass spectrometry (LC-MS). To focus our study on scaffold-dependent disulfide pairing, the achiral glycine residues were preferentially placed in the peptide, which disfavour the sequence-dependent prefolding of the peptide towards specific isomers. The oxidative folding of **1** results in the formation of scrambled isomers that cannot be isolated efficiently using high-performance liquid chromatography (HPLC) (Fig. 2a and b). In addition, the total peak area of the produced isomers is as low as 20% of the initially reduced peptide, suggesting that the overall yield of the folding is rather low, and it is very possible that this involves many undetectable and trapped folding intermediates.

We then rationally and subjectively transformed the cysteine framework of **1** into an artificial one by simultaneously replacing the two adjacent cysteines with a synthetic dithiol amino acid and three of the others with Pen residues,^8,9h^ which generates a new peptide **2** with a PenXC–Pen–Pen–Dtaa framework (Fig. 1 and 2; see the ESI† for details on the synthesis of **2**). We hypothesized that the homogenization of the two adjacent cysteines using Dtaa substitution and the spatial separation of the two thiol groups in Dtaa could in principle exclude the formation of 9 specific isomers from the total of 15 possible ones (Fig. 2a). The three-Pen substitution could further reduce the complexity of the oxidized products, leading to a reduction in the number of possible isomers from 6 to 3 due to the orthogonal features of the Cys–Pen disulfide pairing (Fig. 2a).^9*h*^ In addition, the formation of the intra-CRC motif disulfide bond is strongly disfavoured due to the intrinsic constrain of the bridged structure.^9g,11^ These inferences, taken together, ultimately restrict the maximum number of final folding products to 2. Indeed, we only observed the formation of two expected isomers (in ∼100% yield, and in a ∼5 : 1 ratio of the two peaks) in the HPLC traces after the oxidative folding of **2** in an oxidized glutathione (GSSG) buffer, whereas the other peaks (or isomers) were negligibly small (Fig. 2b). In both of the isomers (**2a** and **2b**; Fig. 2a and c), two of the three Pen residues are paired with the Dtaa dithiol, and with the odd one paired to the sole cysteine residue within the peptide (see the ESI for the characterization of the disulfide pairing; Fig. S1–S3†). The isomer **2a** is more favorably formed compared to **2b**, which is likely to arise from superiority in the change in the folding entropy for **2a**, as sequence-related folding processes are largely absent, and thus **2b** would correspond to a topologically more compact scaffold compared to **2a**.

**Fig. 3:**
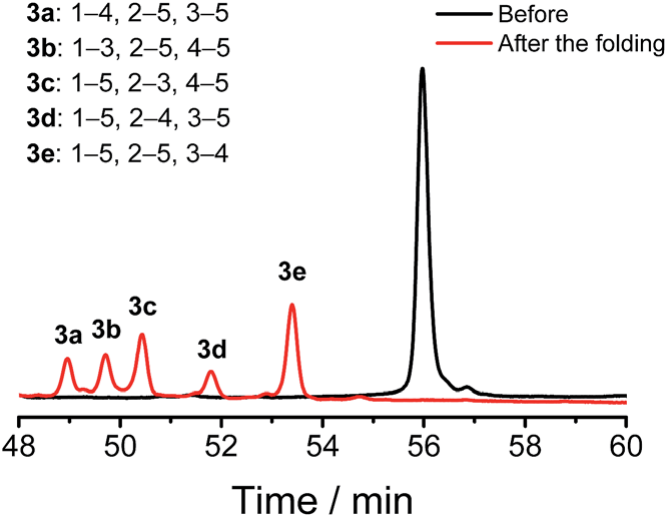
Oxidative folding of **3** (10 µM) in 0.2 mM GSSG and in 50 vol% DMSO/100 mM phosphate buffer (pH 7.4) (black and red line: 0 and 2 h, respectively). Five expected isomers were formed after oxidative folding.

We demonstrated above that Dtaa and Pen substitution in a six-cysteine peptide can dramatically reduce the complexity of the oxidative folding products, that is, from a total of 15 isomers to the 2 that are expected. The effect of Dtaa substitution on the overall reductive isomerization is quite clear in theory,^8^ whereas the parallel effect of Pen might not be as straightforward. To further reveal the contribution of the Cys/Pen substitution on the reductive isomerization, a peptide (**3**, CXC–C–C–Dtaa framework), that is analogous to **2** but without replacing the three cysteines with Pen residues, was designed and synthesized (Fig. 3). Although it is unlike peptide **1**, the oxidation of **3** results in the formation of 5 distinct and highly resolved HPLC peaks which correspond to the 5 expected isomers (except for the 1–2, 3–5, and 4–5 connectivities shown in Fig. 2a, because of the prohibition of forming an intra-CRC disulfide; see the ESI for the characterization of disulfide pairing; Fig. S4–S9†). However, the total peak area is still significantly diminished (∼50% of the initially reduced peptide), implying a relatively low oxidative folding yield (similar to that for **1**). In addition, we found that the most abundant product of **3** (*i.e.*, the bell-shaped isomer **3e**) is not of the same folding as that obtained from **2** (Fig. 3). The two isomers with disulfide connectivities equivalent to those of **2a** and **2b** are **3c** and **3d**, respectively, which are the second to least and the least abundant products of **3**, respectively. This finding suggests that the driving force arising from the orthogonal disulfide pairing between the thiols of cysteine/Dtaa and the sterically hindered thiols of Pen residues can overcome the topology-preferred folding propensity to the bell-shaped isomer and can direct the oxidative folding of the peptide to the two specific and expected isomers.

**Fig. 4:**
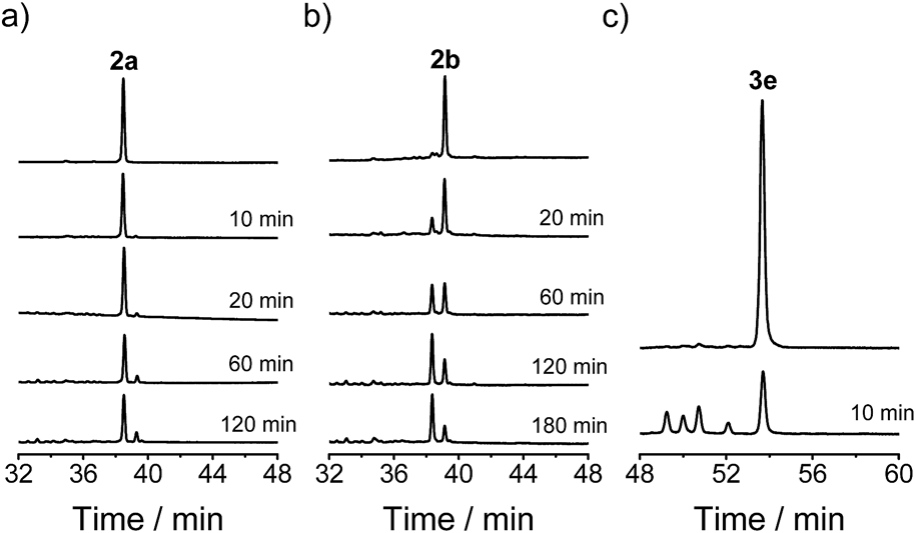
Chromatograms showing the isomerization of **2a** (a), **2b** (b), and **3e** (c) in 0.5 mM GSH/GSSG buffer; peptide concentration: 5 µM. The chromatograms were shifted relative to each other for clarity.

**2a**, **2b** and **3e** were then isolated using HPLC and dissolved in a glutathione (GSH)/GSSG buffer (0.5 mM, pH 7.4). Interestingly, **2a** and **2b** are both substantially more stable than **3e** (Fig. 4). **3e** is subject to very rapid disulfide isomerizations, which result in the formation of the other four isomers and an equilibrium was achieved within 10 min. In contrast, the equilibrium of the disulfide isomerizations for **2a** and **2b** was only achieved after ∼2 h. The enhanced stability is considered to stem from the steric hindrance of the Pen residues (*i.e.*, the two methyl groups adjacent to the sulfur atom). More importantly, side-products were not obviously observed during the disulfide isomerizations (even for the less stable peptide **2b**), suggesting that the oxidative folding pathways used by the artificial six-thiol framework (but not the natural six-cysteine framework or the non-Pen analogue) should have high efficiency and precision towards the expected isomers.

**Fig. 5:**
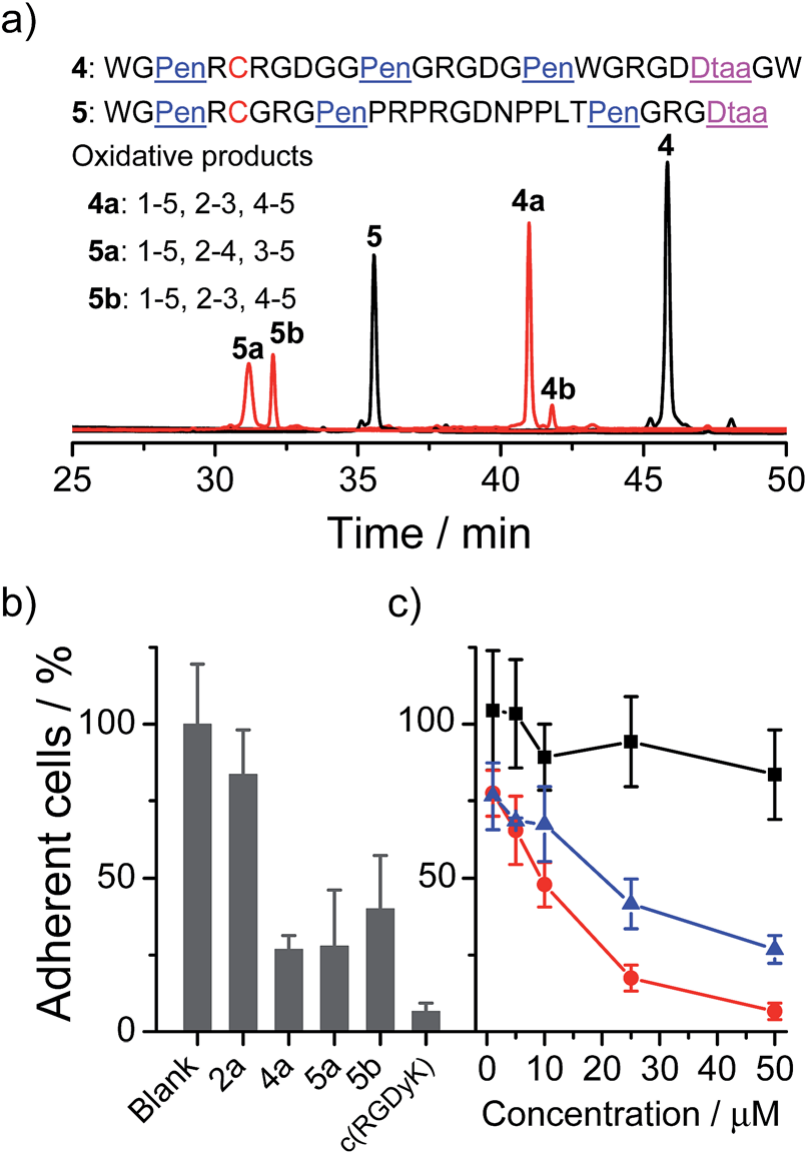
(a) Oxidative folding of peptides **4** (10 µM) in 0.2 mM GSSG and in 50 vol% DMSO/100 mM phosphate buffer (pH 7.4), and **5** (10 µM) in 0.2 mM GSSG and in 100 mM phosphate buffer (pH 7.4) (black and red line: 0 and 2 h, respectively). It is worth noting that oxidized **5** tends to aggregate in buffers, thus **5a** and **5b** cannot be synthesized in large amounts. (b) The inhibition of cell adhesion by the peptides (**2a**, **4a**, **5a**, **5b**, and c(RGDyK) determined by MTT assays; 50 µM); the error bars represent the standard deviation of the mean (*n* ≥ 3). (c) The dose-dependent inhibition of cell adhesion by **2a** (negative control, black line), **4a** (blue line), and c(RGDyK) (positive control, red line); the error bars represent the standard deviation of the mean (*n* ≥ 3).

Next, we examined if the directed oxidative folding of the artificial scaffolds is tolerant to the manipulation of the primary sequence, and if the artificial disulfide-rich scaffold can be used for grafting bioactive sequences. An integrin binding motif RGD (–Arg–Gly–Asp–) was selected for the test.^12^ In the first example, three RGD motifs were inserted into the variable segments (*i.e.*, “–”) of the PenXC–Pen–Pen–Dtaa framework to design peptide **4** (Fig. 5a). Secondly, a yeast-selected sequence (PRPRGDNPPLT;^13^ containing a RGD motif) was grafted into the second variable segment of the framework, and the length of the other two variable segments was shortened by the depletion of two amino acid residues (for the ease of synthesis; **5**, Fig. 5a). Then, the peptides (**4** and **5**) were oxidized under the same conditions as those used for the folding of **2**. As can be seen in Fig. 5a, the oxidative folding of **4** and **5** results in the formation of the expected isomers, with either 1–5, 2–3, 4–5 (**4a** and **5b**) or 1–5, 2–4, 3–5 (**5a**) disulfide connectivity, as the major products (see the ESI for the characterization of disulfide pairing; Fig. S10–S14†), which is very similar to that observed for **2**. We further examined the ability of the oxidized peptides to block the adhesion of U87 glioblastoma cells (with surface-expressed integrin) to cell culture plates. All of the peptides were able to inhibit the adhesion of the cells (Fig. 5b and c). Although the activities of these RGD-containing peptides are lower compared to that of commercially provided cyclic RGD (c(RGDyK); as a positive control), this is not surprising as their cyclic structures have been thoroughly optimized.^12^ In addition, the previously obtained **2a**, as a negative control without an RGD motif, exhibits a negligible ability of inhibiting cell adhesion. We also observed an obvious change in the morphology of the cells on the plates when the cells were incubated with the RGD-containing peptides (**4a**, **5a**, and **5b**; Fig. S18†). Therefore, these results strongly suggested that the developed artificial disulfide-rich scaffolds are tolerant to extensive manipulation in the primary amino acid sequence and are amenable to the design of disulfide-rich bioactive peptides by grafting bioactive sequences.

**Fig. 6:**
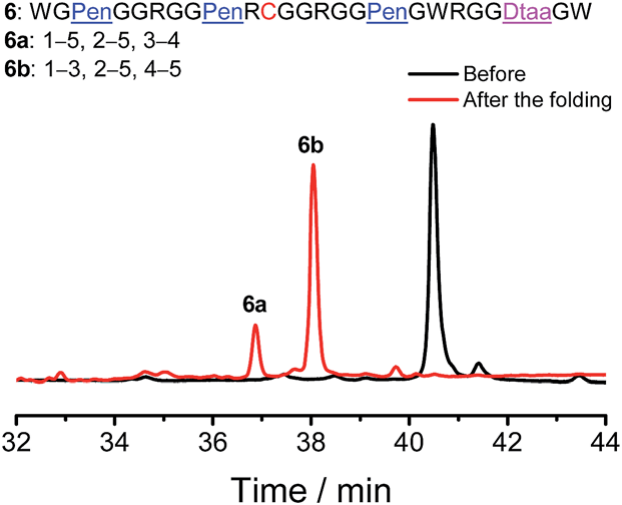
Oxidative folding of **6** (10 µM) in 0.2 mM GSSG and in 50 vol% DMSO/100 mM phosphate buffer (pH 7.4) (black and red line: 0 and 2 h, respectively); the two expected isomers were formed after oxidative folding.

Finally, to demonstrate if our strategy could be used to regulate the folding of peptides with different cysteine frameworks for the design and synthesis of novel artificial disulfide-rich scaffolds, a peptide with a C–CXC–C–CC framework was reengineered using Pen and Dtaa substitution, which generates a new peptide **6** with a unique Pen–PenXC–Pen–Dtaa framework (Fig. 6). We found that the oxidation of **6** in buffers leads to the formation of the two expected isomers (∼100% yield), in which the Pen residues are paired with either the Dtaa dithiol or the cysteine residue (**6a** and **6b**; see the ESI for the characterization of disulfide pairing; Fig. S15–S17†). Thus, this result further validates the robustness and generality of the present strategy for the design and synthesis of artificial peptide scaffolds with precisely defined disulfide patterns and a minimized number of isomers. As the length of the peptide segments in the present scaffolds, the position of the PenXC motif and the Dtaa position can be changed at will, and a number of artificial disulfide-rich scaffolds could be designed and synthesized in the future.

## Conclusions

In conclusion, we developed a combinatorial strategy to regulate the oxidative folding of peptides, which generates a novel class of artificial peptide scaffolds with precisely defined disulfide patterns and a minimized number of isomers. Despite the fact that the folding pathways of natural polypeptides (or proteins) can be strategically tuned through the use of disulfide surrogates or selenocysteine, and that native folds might be obtained in high yields,^9a–c,14^ the precise pairing of disulfide bonds in these systems is considered, in essence, as a result of the primary sequence-specific prefolding or co-folding (*i.e.*, the folding before the disulfide formation or disulfide-directed folding).^6b,15^ It is still inconceivable to fundamentally reduce the complexity of disulfide pairing in peptides containing up to three disulfides without the involvement of sequence-specific folding. In this work, we demonstrated that the total number of isomers formed after the oxidative folding of a peptide containing six thiols can be decreased to a minimum of two (*i.e.*, from 15 to 2). To our knowledge, such elegant precision in peptide folding has never been achieved. As a solely thiol-pattern-based peptide folding strategy, standing out from the existing sequence-dependent ones, it would provide a valuable guide for designing novel bioactive disulfide-rich peptides. Moreover, the artificial disulfide-rich scaffolds have been found to have high stability in redox buffers and should be more able to avoid problematic isomerization due to the presence of fewer isometric structures compared to normal six-cysteine-containing peptides. We believe that artificial disulfide-rich scaffolds with an intrinsic and precise disulfide pairing propensity would be more tolerant to sequence manipulation than natural peptide scaffolds. This feature would greatly benefit the development of structurally constrained and multicyclic peptide therapeutics and ligands.
