# Supplementary material for: Artificial disulfide-rich peptide scaffolds with precisely defined disulfide patterns and a minimized number of isomers
Source: Chem Sci. 2017 Feb 17;8(4):2547–52. doi: 10.1039/c6sc05710a (PMC5431680; doi:10.1039/c6sc05710a)
Supplement: Supplementary file 1 [file SC-008-C6SC05710A-s001.pdf]

Electronic Supplementary Material (ESI) for Chemical Science.
This journal is © The Royal Society of Chemistry 2017

Artificial Disulfide-Rich Peptide Scaffolds with Precisely Defined Disulfide Patterns and Minimized Number of Isomers

Yiwu Zheng, Zhuoru Li, Jing Ren, Weidong Liu, Yaqi Wu, Yibing Zhao, and Chuanliu Wu*

Department of Chemistry, College of Chemistry and Chemical Engineering, State Key Laboratory of Physical Chemistry of Solid Surfaces, The MOE Key Laboratory of Spectrochemical Analysis and Instrumentation, Xiamen University, Xiamen, 361005, P.R. China.

*To whom correspondence should be addressed, Email: chlwu@xmu.edu.cn

Table of contents

Experimental Section……………………………………………………………….…………….3

1) **Materials and Instruments**
2) **Oxidative folding of peptides**
3) **Analysis of disulfide pairing for cyclic peptides**
4) **Scheme S1 | Synthesis of dithiol molecule**
5) **Scheme S2 | Synthesis of peptides 2 and 3**
6) **Cell experiments**

Figure | S1 Chromatogram of the products formed after the oxidation of 2……………………9

Figure | S2 Tryptic digestion LC-MS analysis of 2a..10

Figure | S3 Tryptic digestion LC-MS analysis of 2b……………………………………...11

Figure | S4 Chromatogram of the products formed after the oxidation of 3…………………...13

Figure | S5 Tryptic digestion LC-MS analysis of 3a……………………………………...14

Figure | S6 Tryptic digestion LC-MS analysis of 3b……………………………………...16

Figure | S7 Tryptic digestion LC-MS analysis of 3c……………………………………...18

Figure | S8 Tryptic digestion LC-MS analysis of 3d……………………………………...19

Figure | S9 Tryptic digestion LC-MS analysis of 3e………………………………………22

Figure | S10 Chromatogram of the products formed after the oxidation of 4………………….24

Figure | S11 Tryptic digestion LC-MS analysis of 4a…………………………………….25

Figure | S12 Chromatogram of the products formed after the oxidation of 5………………….26

Figure | S13 Tryptic digestion LC-MS analysis of 5a…………………………………….27

Figure | S14 Tryptic digestion LC-MS analysis of 5b…………………………………….28

Figure | S15 Chromatogram of the products formed after the oxidation of 6………………….29

Figure | S16 Tryptic digestion LC-MS analysis of 6a…………………………………….30

Figure | S17 Tryptic digestion LC-MS analysis of 6b…………………………………….32

Figure | S18.The ability of RGD-containing peptides to block U87 cells adhesion were shown by inverted optical microscope……………………………………………………………..34

Experimental Section

1) Materials and Instruments. The investigated peptides, *N*-terminally acetylated and *C*-terminally amidated, were supplied by GL Biochem (Shanghai) or Sangon Biotech (Shanghai) at >95% purity. All peptides were supplied with analytical chromatograms and mass spectra to confirm the identity and purity. Other chemicals were purchased from major suppliers such as Sigma-Aldrich (Beijing) and J&K Chemical (Guangzhou). U87 cells were purchased from CoBioer Biosciences CO., LTD (Nanjing, China). Dulbecco's Modified Eagle Medium (DMEM) with high glucose and phosphate buffered saline (PBS) were obtained from Thermo Scientific (Beijing, China). Eppendorf tubes (1.5 mL), 24-well chambers and cell culture dishes were purchased from JET BIOFIL (Guangzhou, China). MTT was purchased from Sigma-Aldrich. Ultrapure water was used throughout the experiments. Analytical and semi-preparative HPLC was performed using a SHIMADZU system equipped with a prominence LC-20AD solvent delivery unit, a prominence DGU-20A3R degassing unit, a prominence SIL-20A autosampler, a prominence CTO-20A column oven, and a prominence SPD-M20A photodiode array detector. A HITACHI U-3900H UV/Vis spectrometer was used for measuring peptide concentrations. A Bruker En Apex ultra 7.0T FT-MS and a Bruker Esquire 3000 plus iontrap ESI mass spectrometry were used for identifying isolated peptides and fragments generated from tryptic digestion. NMR spectra (compounds **1** and **2** shown in Scheme S1) were recorded on a 400 MHz Bruker Avance NMR spectrometer. OLYMPUS CKX41 was used for observing the ability of RGD-containg peptides to block U87 cells adhesion.

2) Oxidative folding of peptides. Fully reduced peptides were dissolved in 100 mM phosphate buffer (pH 7.4) containing 50 vol% DMSO and 0.2 mM GSSG to achieve a concentration of ~10 μM. Two hours later, aliquots were taken into an empty tube and were immediately treated with 10% HPO_3 to quench the oxidative reactions. The samples were then analyzed by analytical HPLC (80 μL injection volume, 1.0 mL/min flow rate, isocratic with 5 vol% acetonitrile (0.1% trifluoroacetic acid) for 5 min followed by a linear gradient of acetonitrile (0.1% trifluoroacetic acid) over 90 min or 160min (5–80 vol%).

3) Analysis of disulfide pairing for cyclic peptides. Certain amounts of oxidative peptides (isolated by HPLC, ~1–30 μg) were dissolved in 90 μL phosphate buffer (100 mM, pH = 6), which were then digested by the addition of 10 μL aqueous solution of trypsin (1 mg/mL) at room temperature (~0.5 h). The digested fragments were then analyzed by LC-MS.

4) Synthesis of dithiol molecule

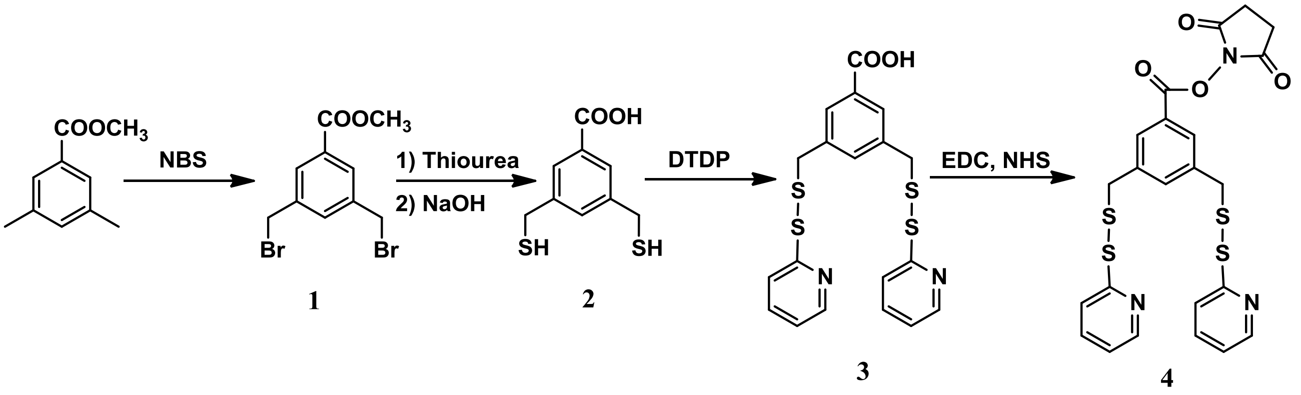

Scheme S1. Synthesis of dithiol molecule.

3,5-Bis(mercaptomethyl)benzoic acid (2) was prepared following a published procedure (Staab, H.A.; Kirrstetter, R.G.H.; *Liebigs Ann. Chem.* **1979**, 886.). Methyl 3,5-dimethylbenzoate (0.6 g, 3.6

mmol), NBS (1.48 g, 8.0 mmol) and benzoyl peroxide (185 mg, 0.72mmol) in 15 mL CCl_4 was refluxed for 4 h. The precipitate was filtered off and washed with hot CCl_4. The filtrate was concentrated and the residue was crystallized from petrol ether, then recrystallized from ether to give a white crystal (**1**; 0.5636 g, 49.1%). ^{1}H NMR (400 MHz, $CDCl_3$) δ 8.09 (d, J = 1.7 Hz, 2H), 7.70 (t, J = 1.8 Hz, 1H), 4.54 (s, 4H), 3.95 (s, 3H).

1 (0.7 g, 2.2 mmol), thiourea (0.42 g, 5.5 mmol) in 10 mL ethanol was refluxed for 6 h. The solvent was evaporated. NaOH (0.5 g, 13.2 mmol) and deoxygened H_2O (20 mL) were added, and the mixture was refluxed under a nitrogen atmosphere for 4 h. The resulting mixture was cooled down to 0 ℃ and neutralized with deoxygened 1 N HCl. The precipitate was collected and washed with H_2O. The crude was recrystallized from ethanol to yield a white solid (0.4274 g, 90.8%). ^{1}H NMR (400 MHz, DMSO-d6) δ 7.79 (d, J = 1.8 Hz, 2H), 7.55 (d, J = 1.9 Hz, 1H), 3.78 (d, J = 7.9 Hz, 4H), 2.96 (t, J = 7.8 Hz, 2H). ESI-MS ($C_9H_9O_2S_2$ $[M-H]^-$, m/z), calcd: 213.00; found: 212.80.

3,5-bis((pyridin-2-yldisulfanyl)methyl)benzoic acid (3). **2** (5.3 mg, 25 μmol) was added to 2,2'-dithiodipyridine (29.7 mg, 0.14 mmol) in 10 mL acetonitrile with 1 mL 50 mM PB (pH =7.4) buffer under a nitrogen atmosphere. The reaction mixture was stirred at ambient temperature for 30min. The mixture was concentrated and purified by reversed-phase HPLC (4.4 mg, 40.7%). HRMS ($C_{19}H_{17}N_2O_2S_4$ $[M+H]^+$, m/z), calcd: 433.0173; found: 433.0175.

2,5-dioxopyrrolidin-1-yl 3,5-bis((pyridin-2-yldisulfanyl)methyl)benzoate (4). EDC·HCl (3.9 mg, 20 μmol) and NHS (2.3 mg, 20 μmol) were added to **3** (4.3 mg, 10 μmol) in DMSO (500 μL).

The mixture was stirred at ambient temperature for 4 h, and the crude was then purified by reversed-phase HPLC (4.5 mg, 85.2%; purity, >95%). ^{1}H NMR (400 MHz, Chloroform-*d*) δ 8.50 (ddd, *J* = 5.0, 1.8, 0.9 Hz, 2H), 7.82 (d, *J* = 1.8 Hz, 2H), 7.68 (ddd, *J* = 8.1, 7.4, 1.8 Hz, 2H), 7.56 (t, *J* = 1.0 Hz, 1H), 7.54 (t, *J* = 1.0 Hz, 1H), 7.52 (t, *J* = 1.7 Hz, 1H), 7.18 (ddd, *J* = 7.4, 5.1, 1.1 Hz, 2H), 4.01 (s, 4H), 2.95 (s, 4H). ESI-MS ($C_{23}H_{20}N_3O_4S_4$, m/z), calcd: 530.03; found: 529.88.

5) Synthesis of peptides 2–6.

compound 4 + WGPenRCGGRGGPenGGRGGPenGWRGGKGW ⟶ WGPenRCGGRGGPenGGRGGPenGWRGGDtaaGW

Peptide **2**

compound 4 + WGCRCGGRGGCGGRGGCGWRGGKGW ⟶ WGCRCGGRGGCGGRGGCGWRGGDtaaGW

Peptide **3**

compound 4 + WGPenRCRGDGGPenGRGDGPenWGRGDKGW ⟶ WGPenRCRGDGGPenGRGDGPenWGRGDDtaaGW

Peptide **4**

compound 4 + WGPenRCGRGPenPRPRGDNPPLPenGRGK ⟶ WGPenRCGRGPenPRPRGDNPPLPenGRGDtaa

Peptide **5**

compound 4 + WGPenGGRGGPenRCGGRGGPenGWRGGKGW ⟶ WGPenGGRGGPenRCGGRGGPenGWRGGDtaaGW

Peptide **6**

Scheme S2. Synthesis of peptides **2**–**6**

Peptide **2** (5 mg, 1.98 μmol) dissolved in 500 μL DMSO. 2,2'-Dithiodipyridine (4.4 mg, 20 μmol) was added to the mixture. 1 h later, 4 (1 mg, 1.98 μmol) and 200 mM DIEPA (10 μL) were added. After another 1 h, the reaction was completed, 100 mM TCEP (50 μL) were sequentially added. The reaction was then purified by analytical HPLC (1.0 mL/min flow rate, isocratic with 5 vol% acetonitrile (0.1% trifluoroacetic acid) for 5 min followed by a linear gradient of acetonitrile (0.1% trifluoroacetic acid) over 40 min (5–95 vol%). The peptide was characterized by LC-MS. Calcd (M):

2805.34; found ([M+4H$^+$]$^{4+}$): 702.0. Peptides **3**, **4**, **5** and **6** were synthesized using similar procedure for the synthesis of peptide **2**, which were characterized by LC-MS. **3**: Calcd (M): 2721.19; found ([M+4H$^+$]$^{4+}$): 681.0; **4**: Calcd (M): 2979.45; found ([M+3H$^+$]$^{3+}$): 993.7; **5**: Calcd (M): 2980.16; found ([M+3H$^+$]$^{3+}$): 746.20; **6**: Calcd (M): 2805.34; found ([M+4H$^+$]$^{4+}$): 702.0;

6) Cell culture

U87 cells were maintained in DMEM medium (high glucose) supplemented with 10 % FBS， and 1% *v/v* penicillin/streptomycin（penicillin: 10,000 U·mL^{-1}, streptomycin: 10,000 U·mL^{-1}）at 37 °C in a humidified atmosphere containing 5% CO_2. Cells were passaged at about 80% cell confluency using a 0.25 % trypsin solution.

7) Integrin-dependent cell adhesion assay

Cell adhesion assays were evaluated by MTT. U87 cells were seeded in 24-well plates at an initial cell density of 80000 cells per well and grown overnight at 37 ℃, 5% CO_2. On the second day, the supernate was removed and several different concentrations of peptides were added to each well in 300 μL DMEM contained FBS, incubated at 37 ℃, 5% CO_2 for 3 h. After that, the medium was removed and cells were washed three times with PBS to remove the detached cells. Then 300 μL fresh DMEM and 30 μL MTT (50 mg / mL) was added to each well. The cells were incubated for 4 h at 37 ℃ in culture hood. After that, remove the supernate and add 300 μl MTT solvent (DMSO). Cover with tinfoil and agitate cells on orbital shaker for 15 min. The absorbance was then measured at 490 nm using an ELIASA reader (PerkinElmer Enspire®). The obtained absorbance was blank-

corrected (blank: DMEM+MTT, no cells) and the cell viability in percent was calculated according to the following equation:

$$\text{Cell viability} = (OD_{490}\ \text{sample} / OD_{490}\ \text{control}) \times 100\ \%$$

where OD_{490} sample represents the optical density of the cells treated with peptides and OD_{490} control is the cells only treated with DMEM.

a)

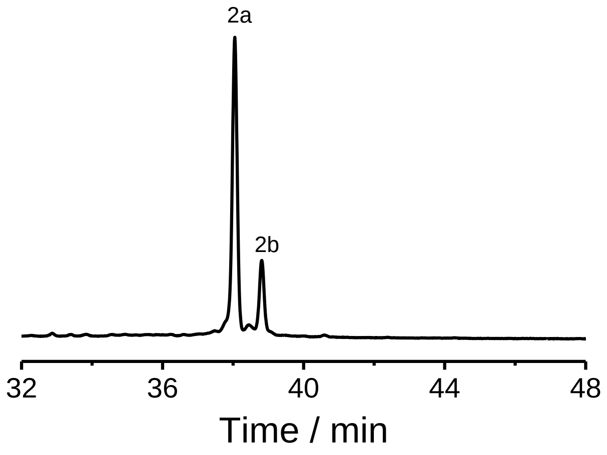

b)

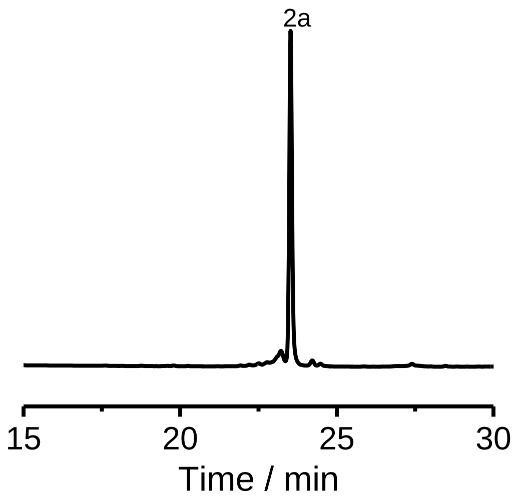

c)

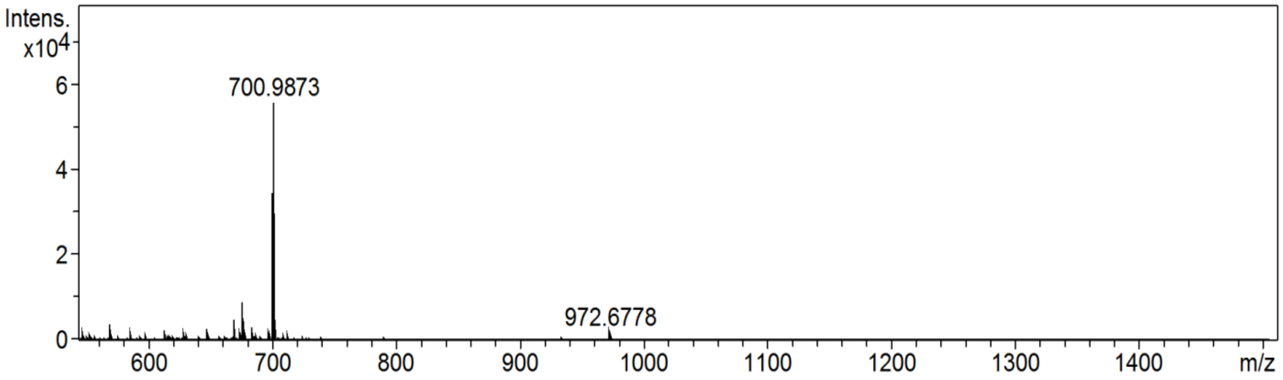

Figure S1. a) Chromatogram of the products formed after the oxidation of **2**. HPLC (1.0 mL/min flow rate, isocratic with 5 vol% acetonitrile (0.1% trifluoroacetic acid) for 5 min followed by a linear gradient of acetonitrile (0.1% trifluoroacetic acid) over 80 min (5–95 vol%). b) Chromatogram of **2a** isolated by HPLC. HPLC (1.0 mL/min flow rate, isocratic with 5 vol% acetonitrile (0.1% trifluoroacetic acid) for 5 min followed by a linear gradient of acetonitrile (0.1% trifluoroacetic acid) over 40 min (5–95 vol%). c) Mass spectrum of **2a**; calcd (m/z): 2799.34; found: ($[M+4H+]^{4+}$): 700.9873.

a)

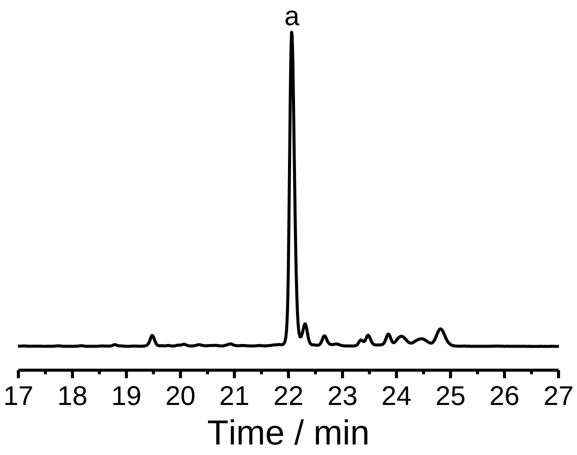

b)

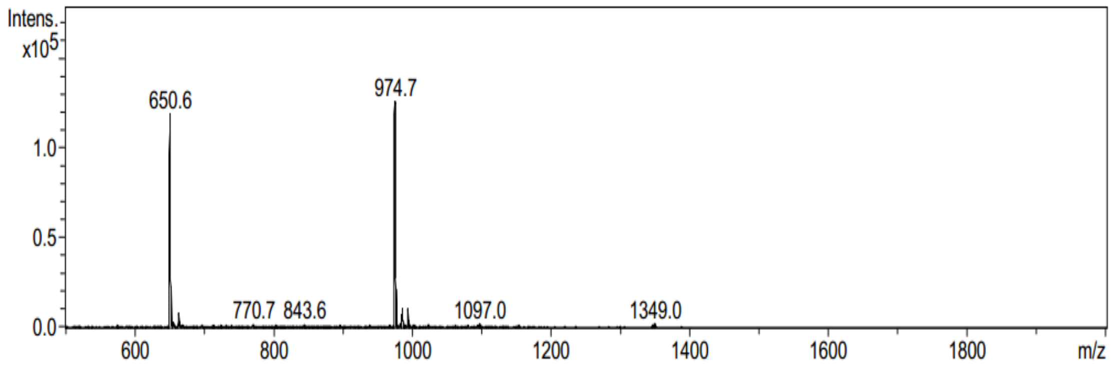

Figure S2. Tryptic digestion LC-MS analysis of **2a**: a) chromatogram of digested fragments from **2a**; b) mass spectra of fragment a labeled in the chromatogram.

Fragment analysis:

**Peak NO.**	**Disulfide pairing**	***m/z*(M+H)$^{+}$ expected**	***m/z*(M+2H)$^{+}$or(M+3H)$^{3+}$ found**
a	1-5, 4-5	1950.24	974.7/650.6

The arrow indicates the cleavage site of trypsin digestion.

Ac-WGPenR ↓ CGGR ↓ GGPenGGR ↓ GGPenGWR ↓ GGDtaaGW-NH$_2$

1 2 3 4 5

a)

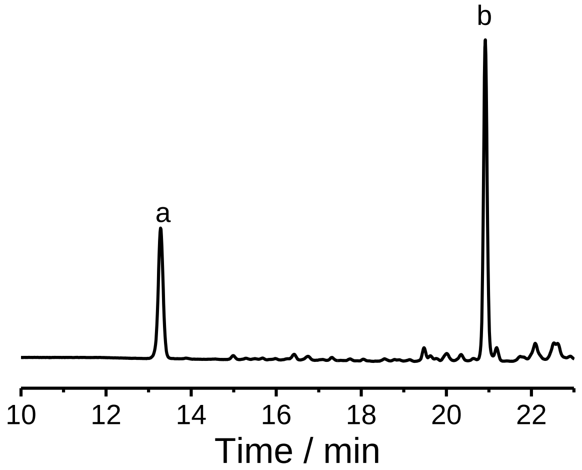

b)

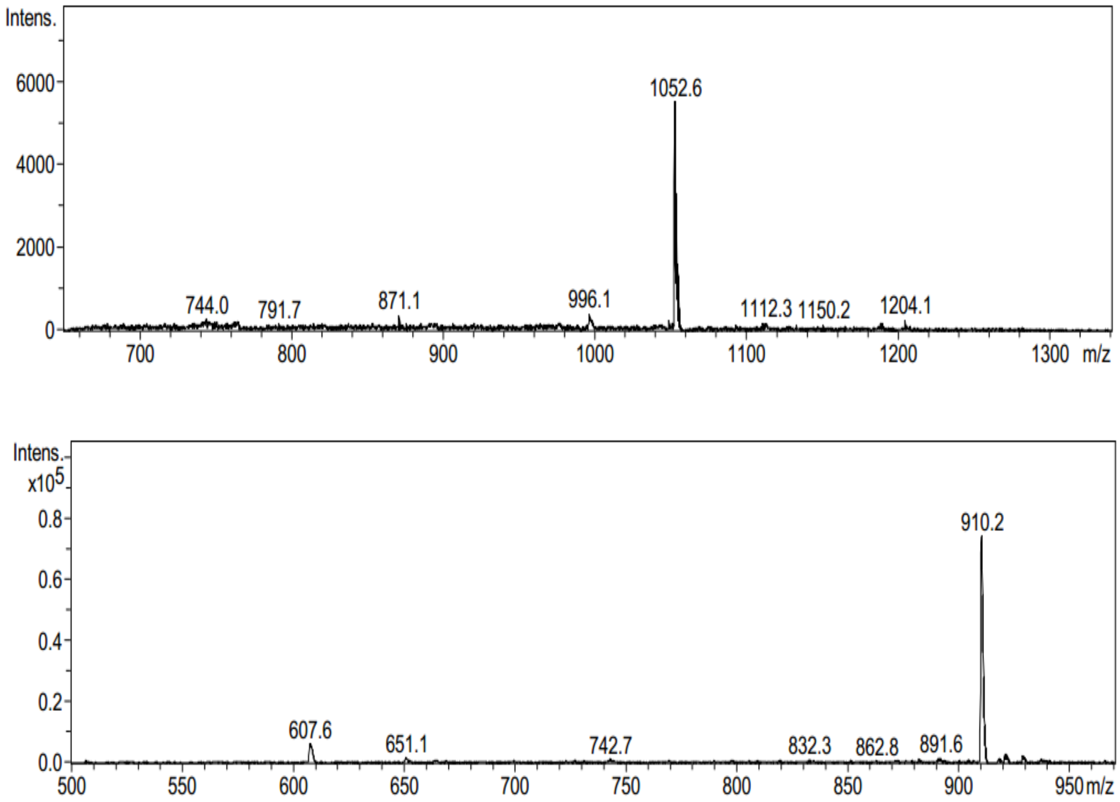

Figure S3. Tryptic digestion LC-MS analysis of **2b**: a) chromatogram of digested fragments from **2b**; b) mass spectra of fragments a and b labeled in the chromatogram.

Fragment analysis:

Peak NO.	Disulfide pairing	*m/z*$(M+2H)^{+}$ expected	*m/z*$(M+2H)^{+}$or$(M+2H)^{2+}$ found
a	2-4	1052.17	1052.6
b	1-5, 3-5	1821.08	910.2

The arrow indicates the cleavage site of trypsin digestion.

Ac-WGPenR ↓ CGGR ↓ GGPenGGR ↓ GGPenGWR ↓ GGDtaaGW-NH_2

(1 = Pen, 2 = C, 3 = Pen, 4 = Pen, 5 = Dtaa)

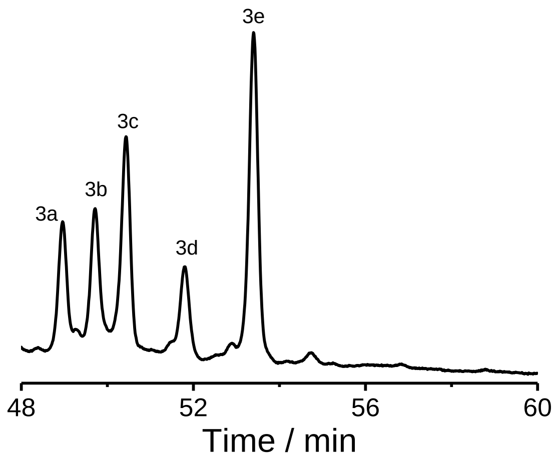

Figure S4. Chromatogram of the products formed after the oxidation of **3**.

a)

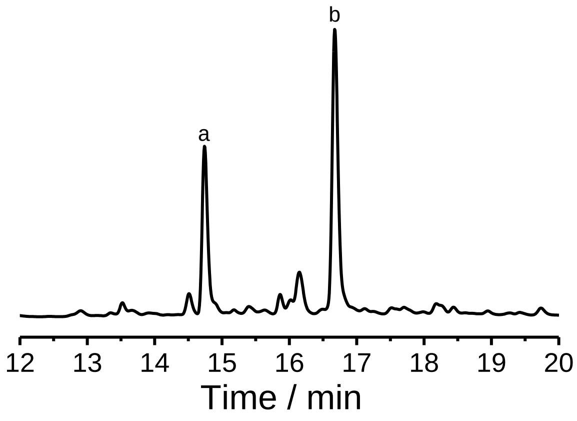

b)

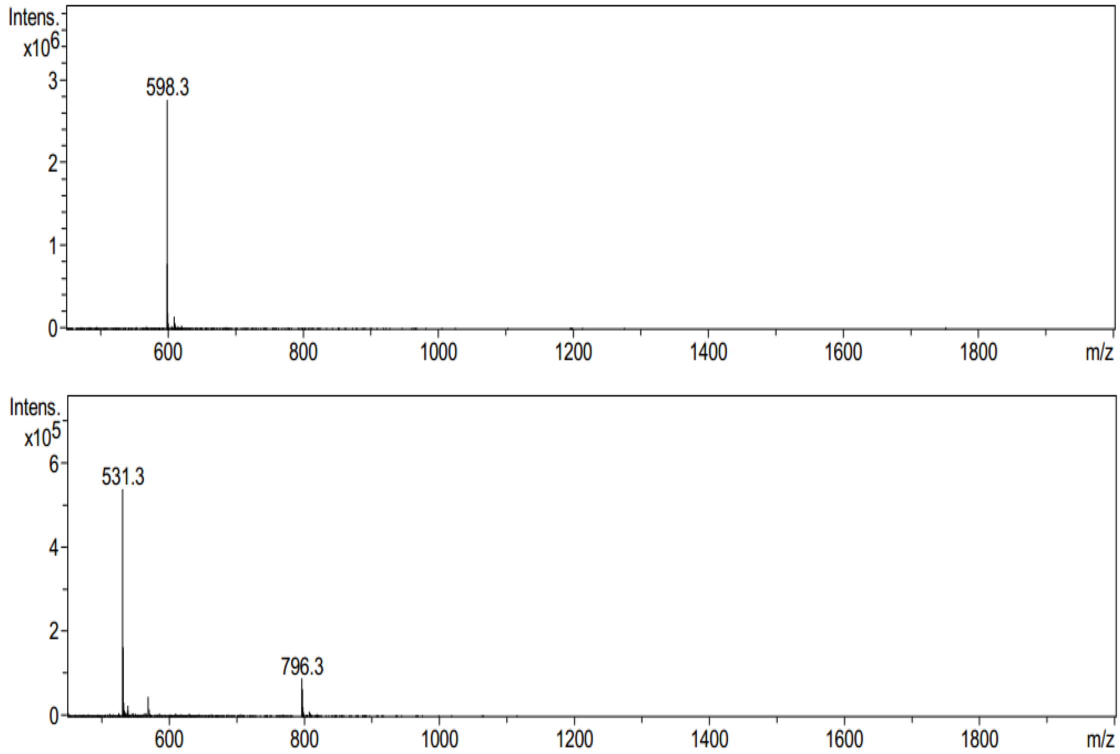

Figure S5. Tryptic digestion LC-MS analysis of **3a**: a) chromatogram of digested fragments from **3a**; b) mass spectra of fragments a and b labeled in the chromatogram.

Fragment analysis:

Peak NO.	Disulfide pairing	*m/z*$(M+2H)^{+}$ expected	*m/z*$(M+2H)^{2+}$or$(M+3H)^{3+}$ found
a	1-4	1197.37	598.3
b	2-5, 3-5	1593.88	796.3/531.3

The arrow indicates the cleavage site of trypsin digestion.

Ac-WGCR ↓ CGGR ↓ GGCGGR ↓ GGCGWR ↓ GGDtaaGW-NH_2

1 2 3 4 5

a)

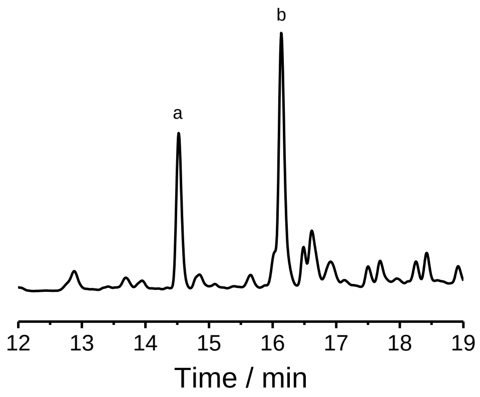

b)

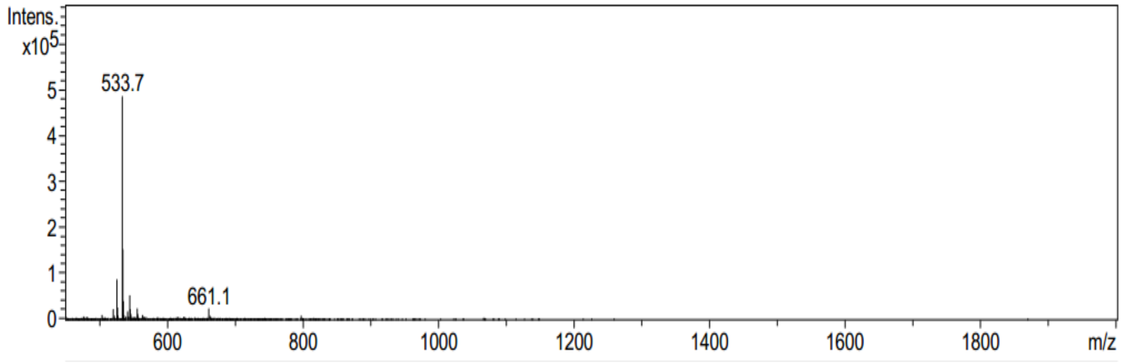

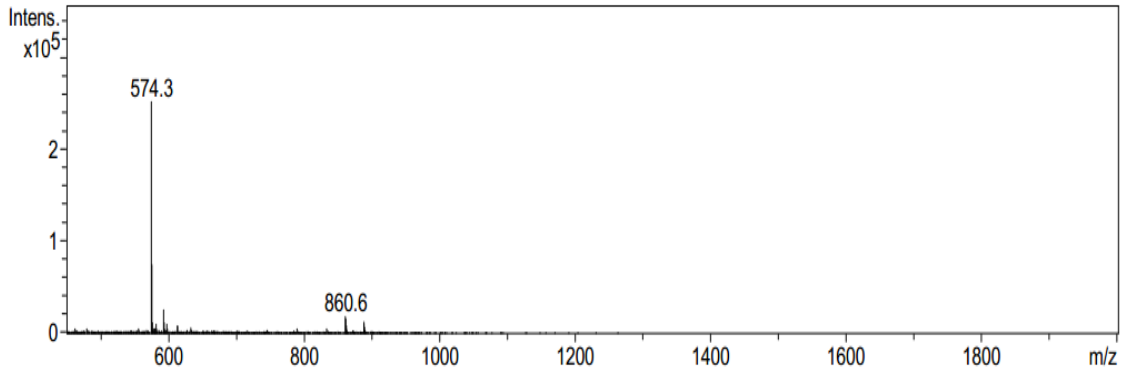

Figure S6. Tryptic digestion LC-MS analysis of **3b**: a) chromatogram of digested fragments from isomer **3b**; b) mass spectra of fragments a and b labeled in the chromatogram.

Fragment analysis:

Peak NO.	Disulfide pairing	$m/z(M+2H)^{+}$ expected	$m/z(M+2H)^{2+}$or$(M+3H)^{3+}$ found
a	1-3	1068.21	533.7
b	2-5, 4-5	1723.04	860.6/574.3

The arrow indicates the cleavage site of trypsin digestion.

Ac-WGCR ↓ CGGR ↓ GGCGGR ↓ GGCGWR ↓ GGDtaaGW-NH_2

1 2 3 4 5

a)

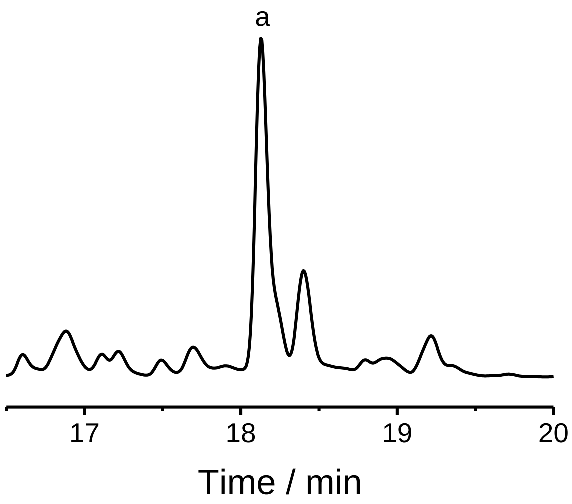

b)

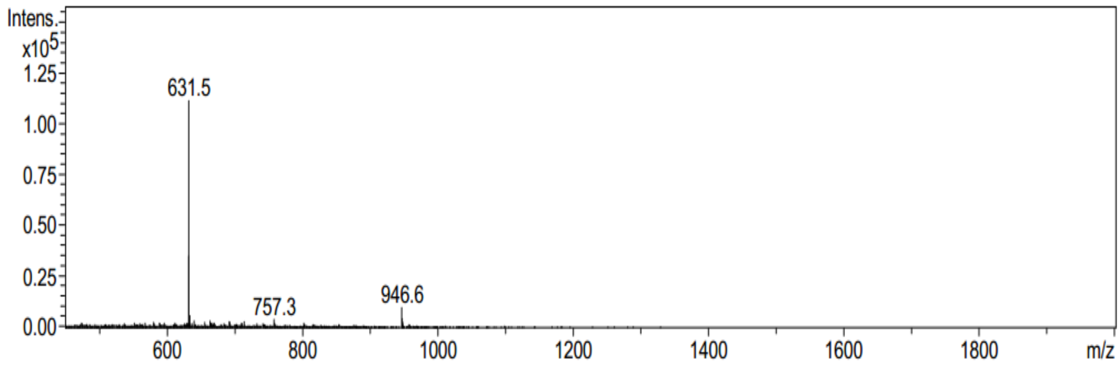

Figure S7. Tryptic digestion LC-MS analysis of **3c**: a) chromatogram of digested fragments from **3c**; b) mass spectra of fragment a labeled in the chromatogram.

Fragment analysis:

Peak NO.	Disulfide pairing	*m/z*(M+2H)$^{+}$ expected	*m/z*(M+2H)$^{2+}$or(M+3H)$^{3+}$ found
a	1-5, 4-5	1894.24	946.6/631.6

The arrow indicates the cleavage site of trypsin digestion.

Ac-WGCR ↓ CGGR ↓ GGCGGR ↓ GGCGWR ↓ GGDtaaGW-NH2

1 2 3 4 5

a)

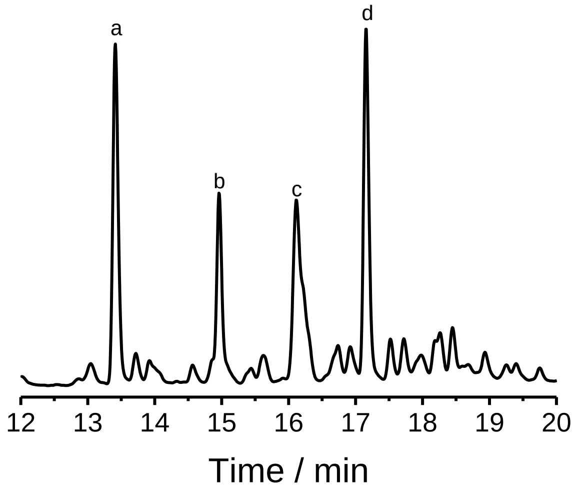

a
b
c
d
12 13 14 15 16 17 18 19 20
Time / min

b)

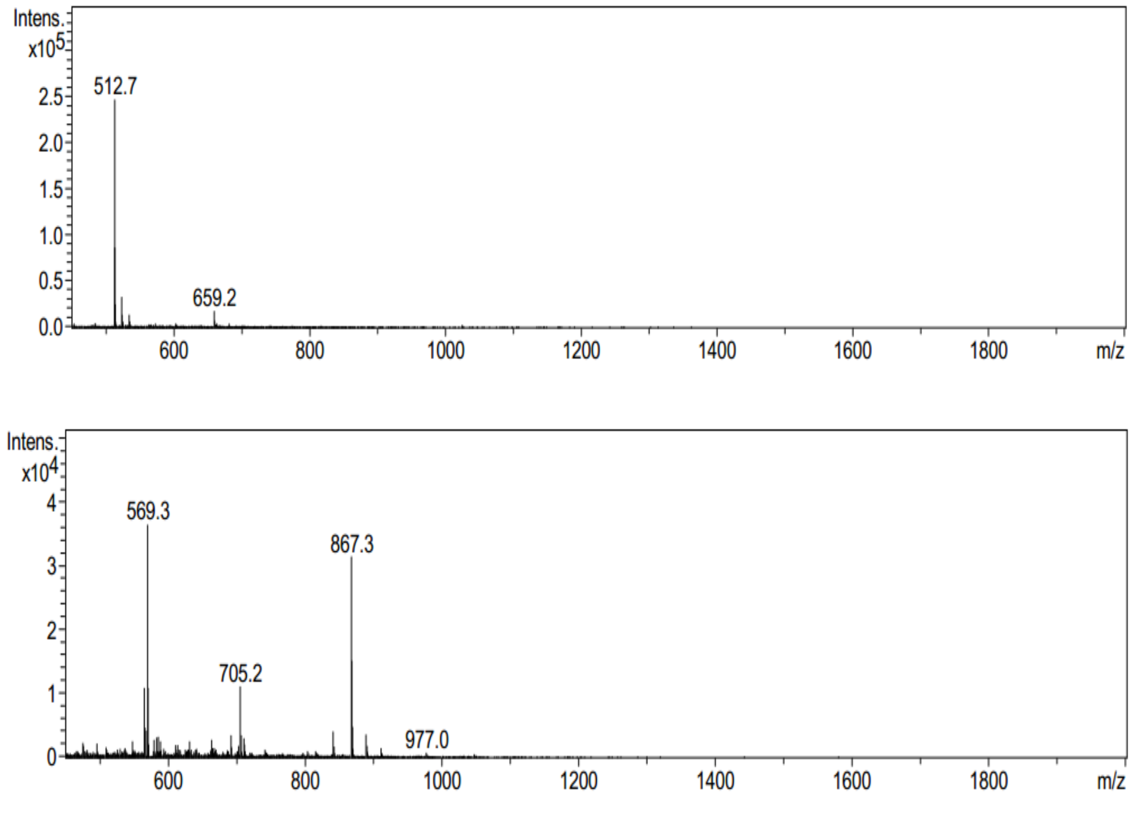

Intens.
x10^5
2.5 2.0 1.5 1.0 0.5 0.0
512.7
659.2
600 800 1000 1200 1400 1600 1800 m/z
Intens.
x10^4
4 3 2 1 0
569.3
867.3
705.2
977.0
600 800 1000 1200 1400 1600 1800 m/z

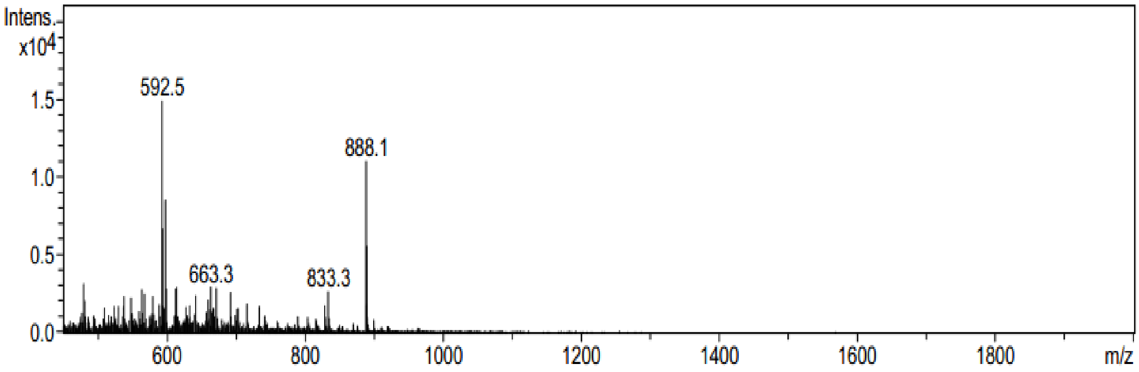

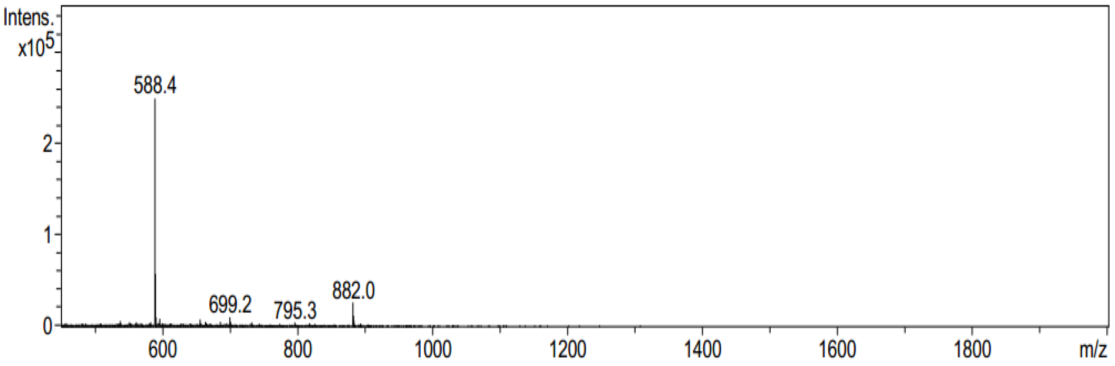

Figure S8. Tryptic digestion LC-MS analysis of **3d**: a) chromatogram of digested fragments from **3d**; b) mass spectra of fragments a–d labeled in the chromatogram.

Fragment analysis:

Peak NO.	Disulfide pairing	*m/z*(M+2H)$^{+}$ expected	*m/z*(M+2H)$^{2+}$or(M+3H)$^{3+}$ found
a	2-4	1026.7	512.7
b	unknown peak	/	867.3
c	unknown peak	/	888.1/592.1
d	1-5, 3-5	1765.08	882.0/588.4

The arrow indicates the cleavage site of trypsin digestion.

Ac-WGCR ↓ CGGR ↓ GGCGGR ↓ GGCGWR ↓ GGDtaaGW-NH_2

1 2 3 4 5

a)

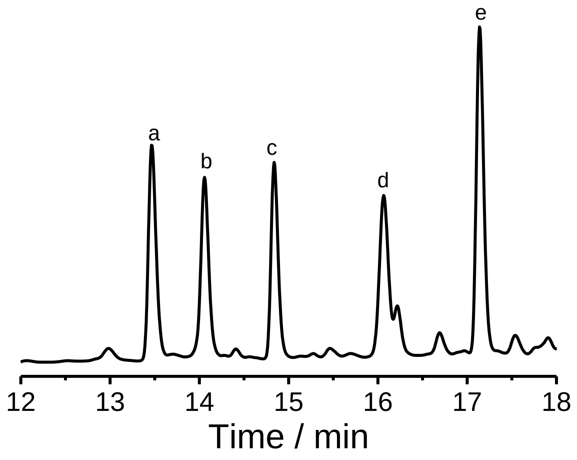
a
b
c
d
e
12
13
14
15
16
17
18
Time / min

b)

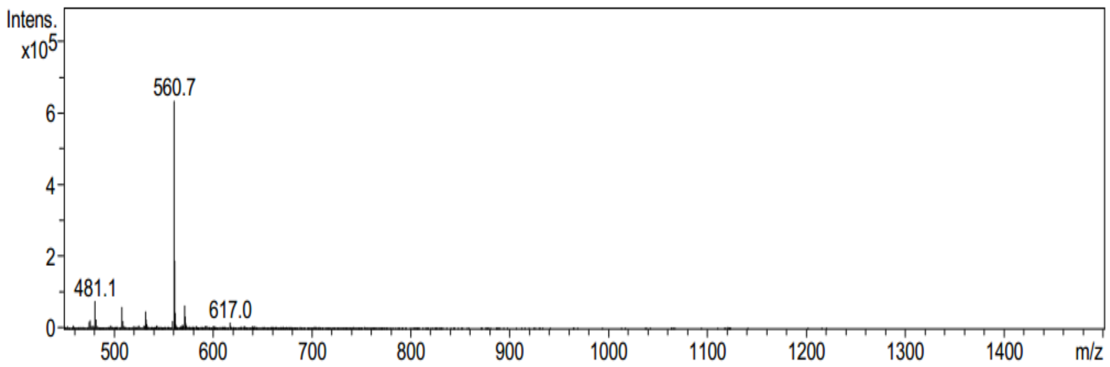
Intens.
x10^5
560.7
6
4
2
0
481.1
617.0
500
600
700
800
900
1000
1100
1200
1300
1400
m/z

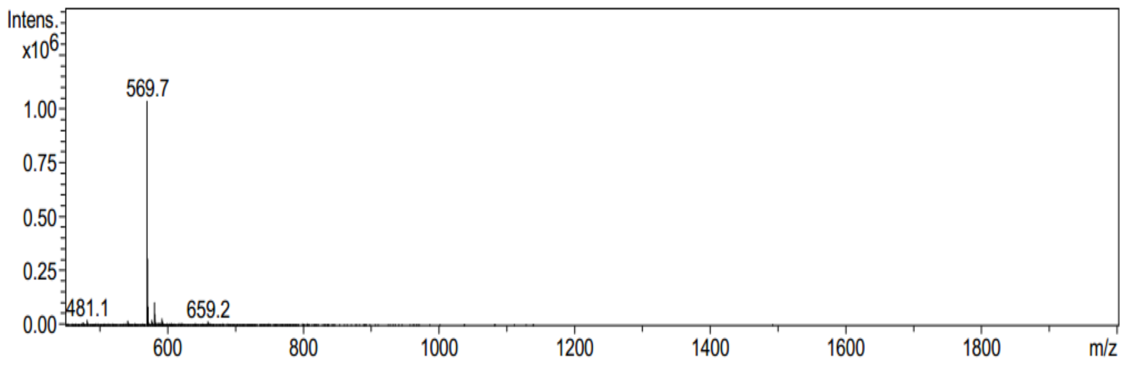
Intens.
x10^6
569.7
1.00
0.75
0.50
0.25
0.00
481.1
659.2
600
800
1000
1200
1400
1600
1800
m/z

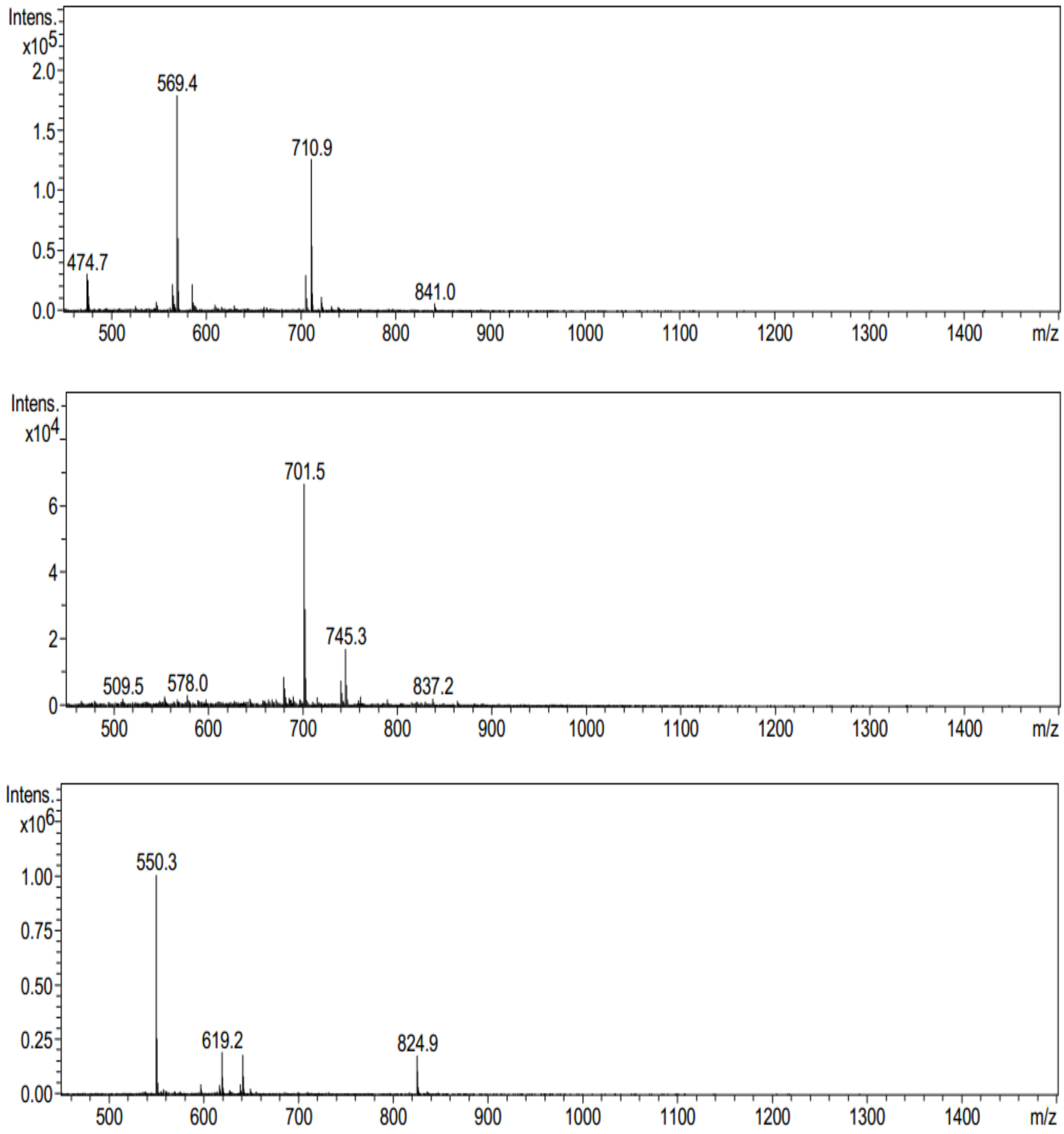

Figure S9. Tryptic digestion LC-MS analysis of **3e**: a) chromatogram of digested fragments from isomer **3e**; b) mass spectra of fragments a–e labeled in the chromatogram.

Fragment analysis:

**Peak NO.**	**Disulfide pairing**	***m/z*(M+2H)$^{+}$ expected**	***m/z*(M+2H)$^{2+}$or(M+3H)$^{3+}$ found**
a	3-4	1140.28	569.7
b*	3-4	1122.28	560.7
c	unknown peak	/	710.5
d	unknown peak	/	701.5
e	1-5, 2-5	1650.97	824.9 / 550.3

b*: the cleavage site within the GGCGGRGGCGWR fragment was not cleaved.

Ac-WGCR ↓ CGGR ↓ GGCGGR ↓ GGCGWR ↓ GGDtaaGW-NH2
1 2 3 4 5

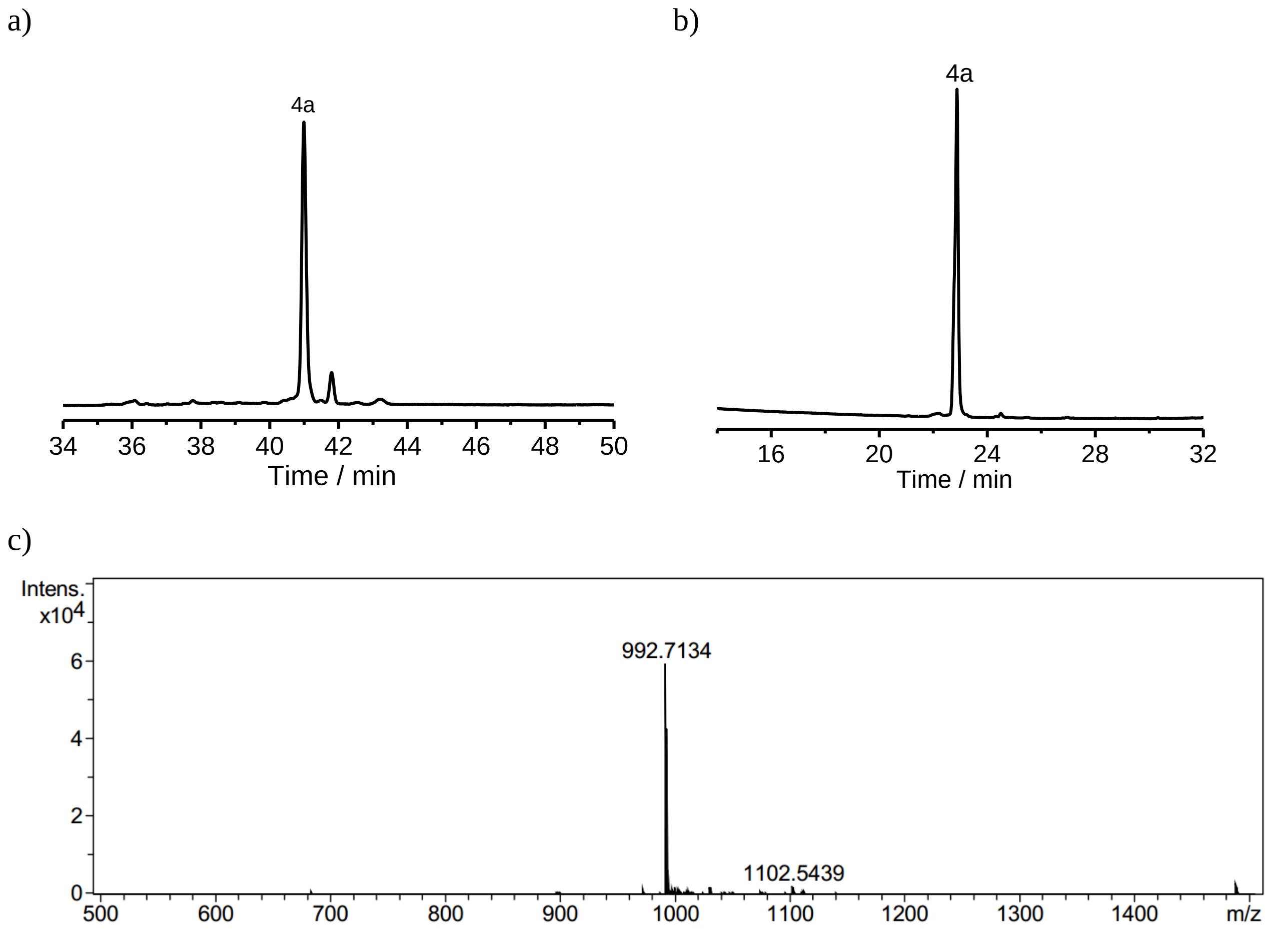

Figure S10. a) Chromatogram of the products formed after the oxidation of **4**. HPLC (1.0 mL/min flow rate, isocratic with 5 vol% acetonitrile (0.1% trifluoroacetic acid) for 5 min followed by a linear gradient of acetonitrile (0.1% trifluoroacetic acid) over 80 min (5–95 vol%). b) Chromatogram of **4a** isolated by HPLC. HPLC (1.0 mL/min flow rate, isocratic with 5 vol% acetonitrile (0.1% trifluoroacetic acid) for 5 min followed by a linear gradient of acetonitrile (0.1% trifluoroacetic acid) over 40 min (5–95 vol%). c) Mass spectrum of **4a**; calcd (m/z): 2973.45; found: ($[M+4H+]^{4+}$): 992.7134.

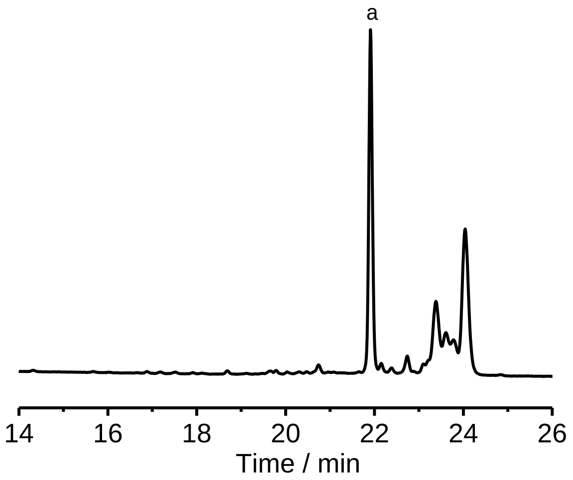

b)

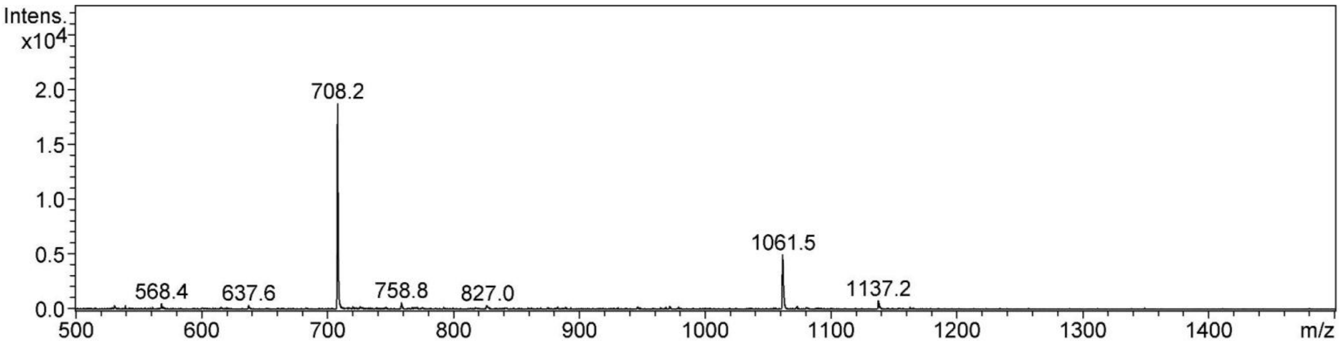

Figure S11. Tryptic digestion LC-MS analysis of **4a**: a) chromatogram of digested fragments from isomer **4a**; b) mass spectra of fragments a labeled in the chromatogram.

Fragment analysis:

Peak NO.	Disulfide pairing	$m/z(M+2H)^{2+}$ expected	$m/z(M+2H)^{2+}$or$(M+3H)^{3+}$ found
a	1-5, 4-5	1061.53	1061.5/708.2

Ac-WGPenR ↓ CR ↓ GDGGPenGR ↓ GDGPenGWGR ↓ GDDtaaGW-NH$_2$
1 2 3 4 5

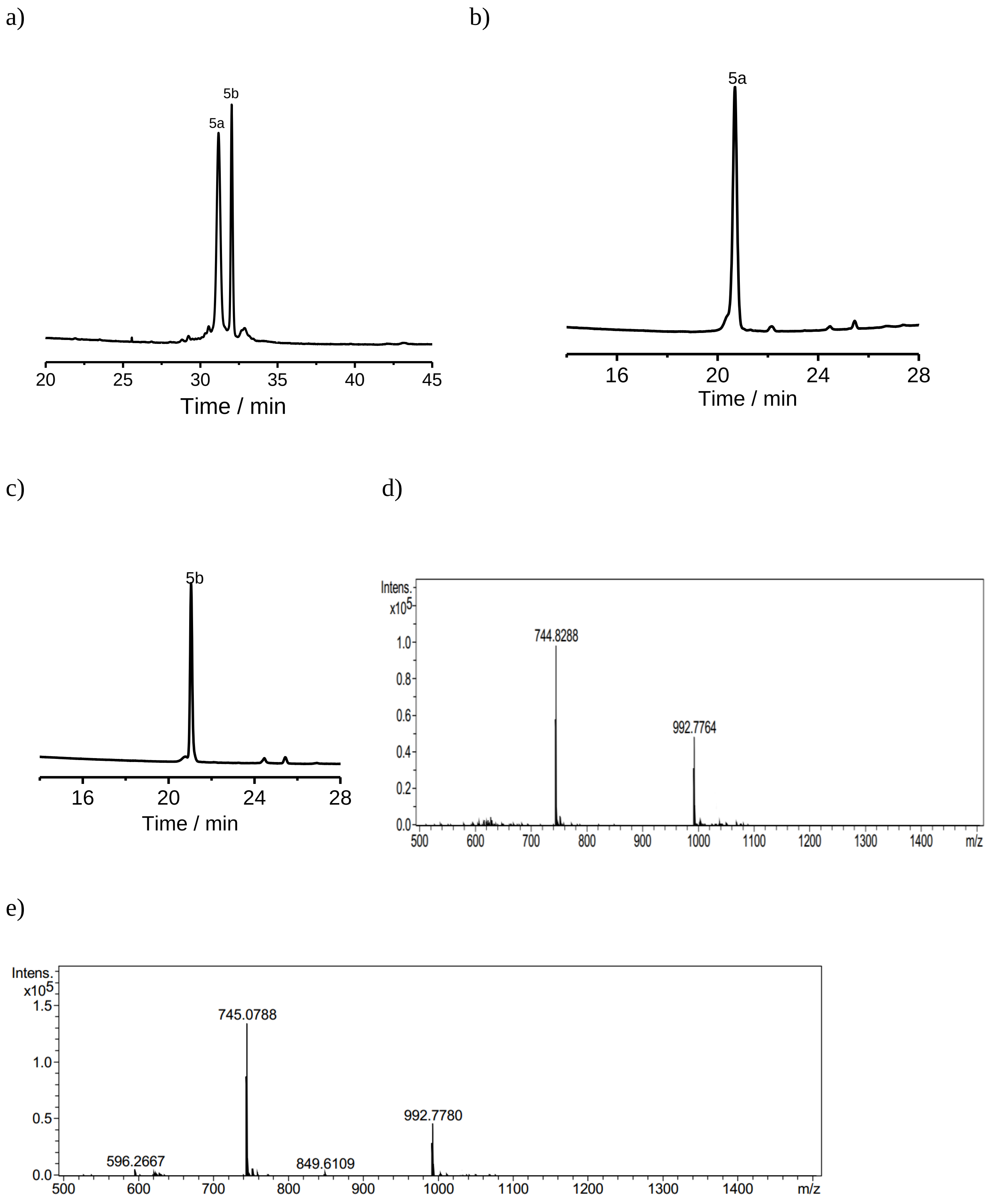

Figure S12. a) Chromatogram of the products formed after the oxidation of **5**. b) Chromatogram of **5a** isolated by HPLC. c) Chromatogram of **5b** isolated by HPLC. d) Mass spectrum of **5a**; calcd (m/z): 2974.16; found: ($[M+4H^+]^{4+}$): 744.8288, ($[M+3H^+]^{3+}$): 992.7764. e) Mass spectrum of **5b**; calcd (m/z): 2974.16; found: ($[M+4H^+]^{4+}$): 745.0788, ($[M+3H^+]^{3+}$): 992.7780.

a)

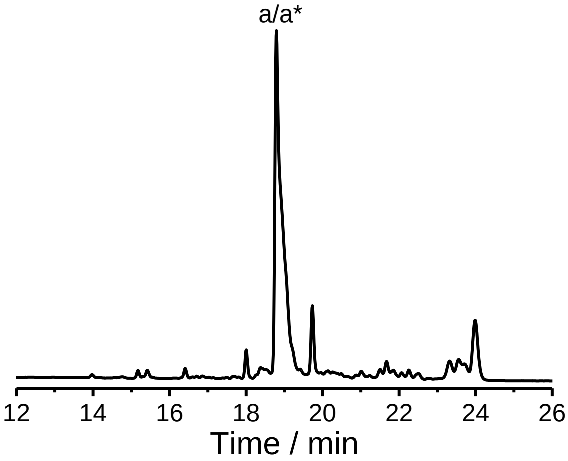

b)

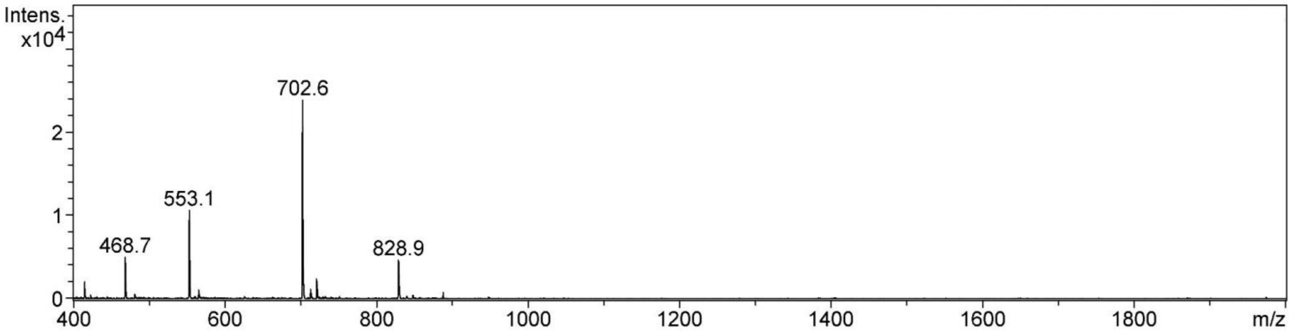

Figure S13. Tryptic digestion LC-MS analysis of **5a**: a) chromatogram of digested fragments from isomer **5a**; b) mass spectra of fragments a labeled in the chromatogram.

Fragment analysis:

Peak NO.	Disulfide pairing	*m/z*(M+2H)$^{2+}$ expected	*m/z*(M+2H)$^{2+}$or(M+3H)$^{3+}$ found
a	1-5, 3-5	702.83	702.6/4683.7
a*	1-5, 3-5	829.48	828.9/553.1

a*: the cleave site within the GPenPRPR fragment were not cleave.

WGPenR ↓ CGR ↓ GPenPR ↓ PR ↓ GDNPPLTPenGR ↓ GDtaa

1 2 3 4 5

a)

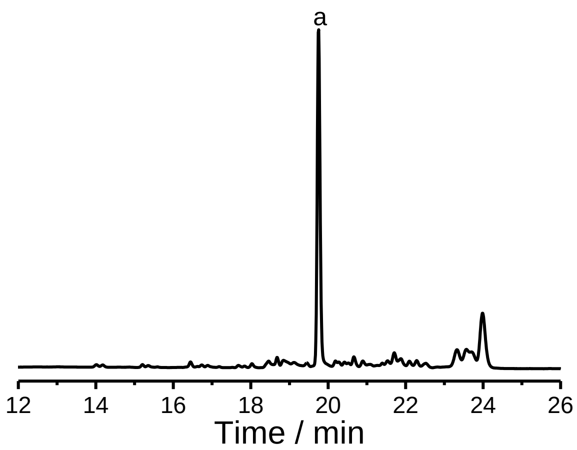

b)

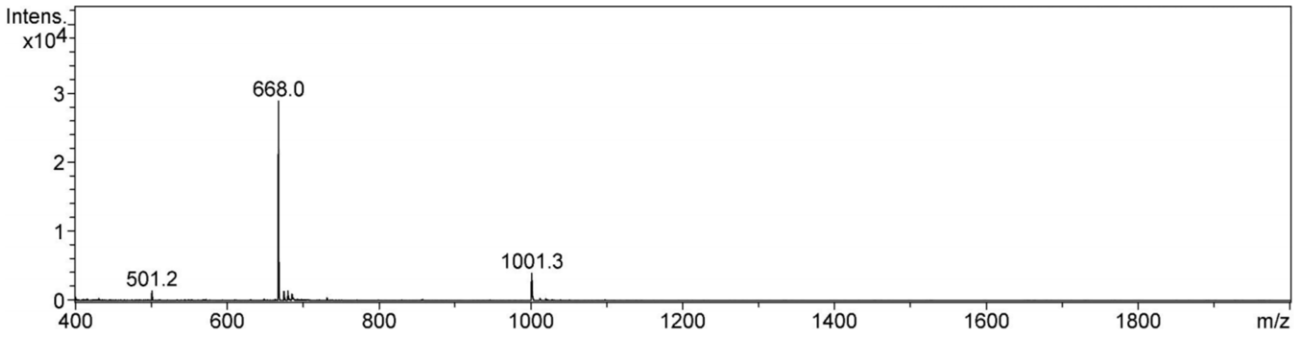

Figure S14. Tryptic digestion LC-MS analysis of **5b**: a) chromatogram of digested fragments from isomer **5b**; b) mass spectra of fragments a labeled in the chromatogram.

Fragment analysis:

Peak NO.	Disulfide pairing	*m/z*$(M+2H)^{2+}$ expected	*m/z*$(M+2H)^{2+}$or$(M+3H)^{3+}$ found
a	1-5, 4-5	1001.57	1001.3/668.0

WGPenR ↓ CGR ↓ GPenPR ↓ PR ↓ GDNPPLTPenGR ↓ GDtaa

1 2 3 4 5

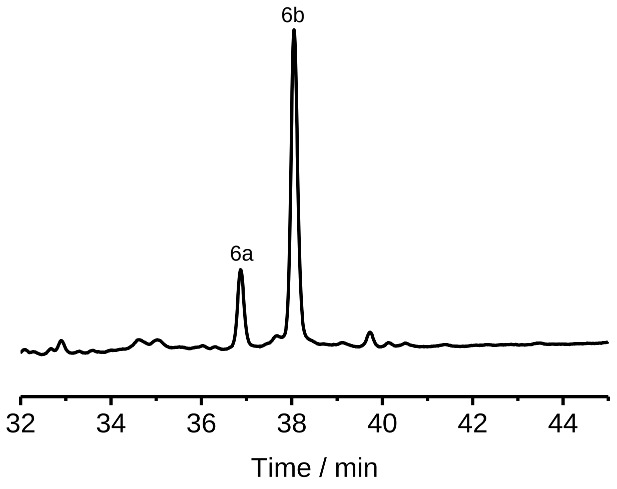

Figure S15. Chromatogram of the products formed after the oxidation of **6**.

a)

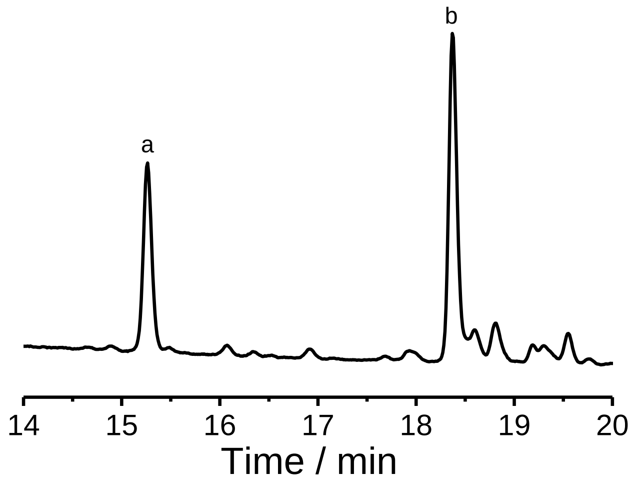

b)

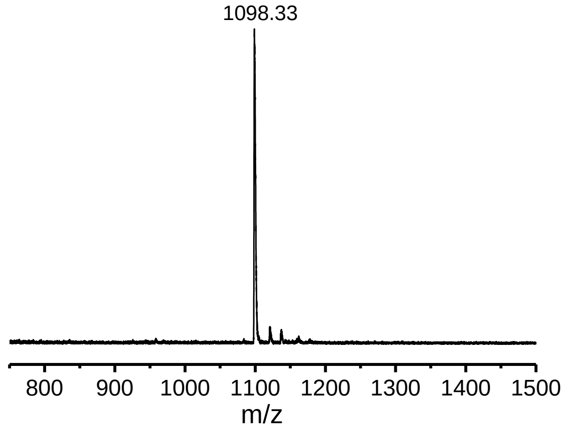

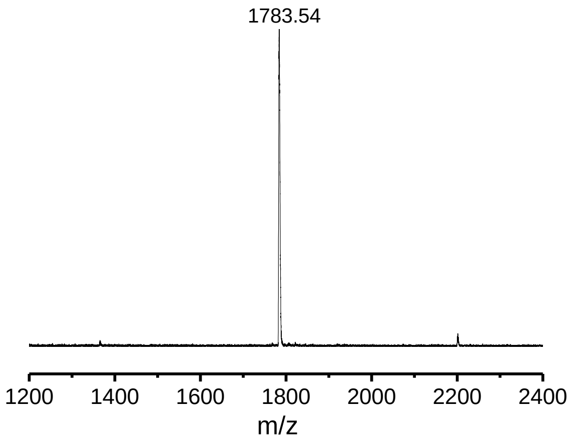

Figure S16. Tryptic digestion HPLC and MALDI-TOF analysis of **6a**: a) chromatogram of digested fragments from isomer **6a**; b) mass spectra of fragments a and b labeled in the chromatogram.

Fragment analysis:

Peak NO.	Disulfide pairing	m/z(M+H)$^{+}$ expected	m/z(M)$^{+}$ found
a	3-4	1095.20	1098.33
b	1-5, 2-5	1777.74	1783.54

Ac-WGPenGGR ↓ GGPenR ↓ CGGR ↓ GGPenGWR ↓ GGDtaaGW-NH_2

1 2 3 4 5

a)

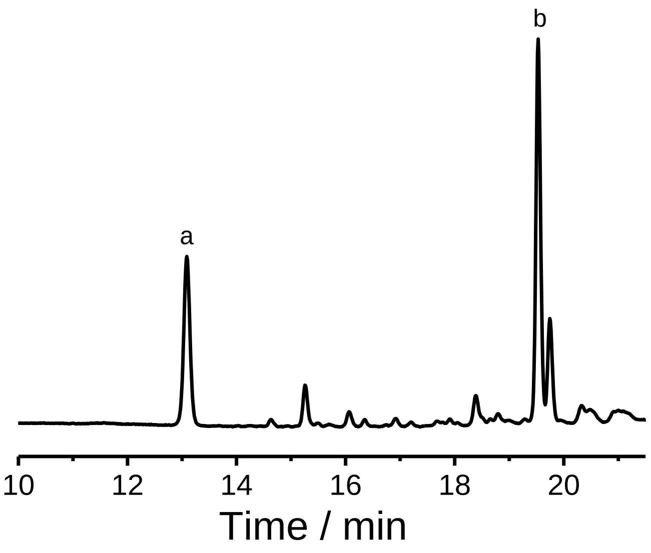

b)

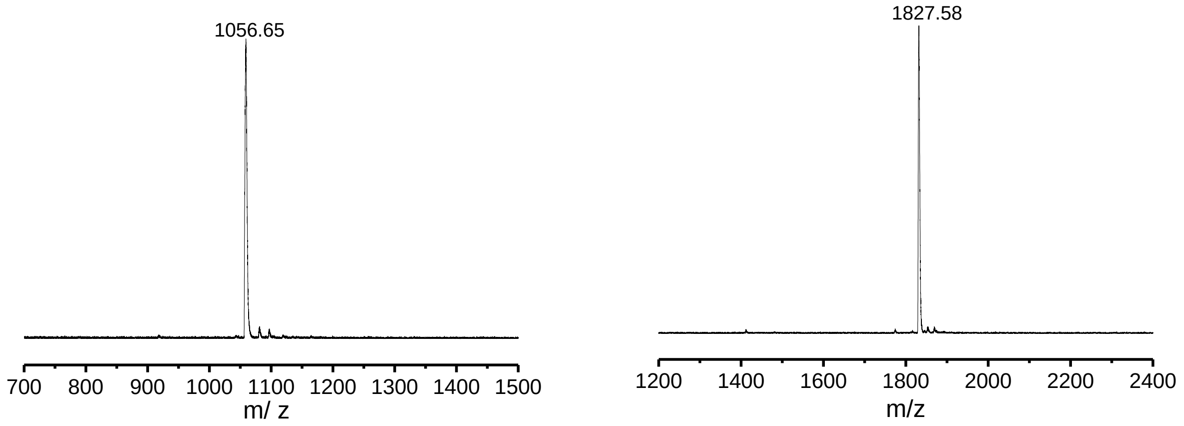

Figure S17. Tryptic digestion HPLC and MALDI-TOF analysis of **6b**: a) chromatogram of digested fragments from isomer **6b**; b) mass spectra of fragments a and b labeled in the chromatogram.

Fragment analysis:

Peak NO.	Disulfide pairing	*m/z*(M+H)$^+$ expected	*m/z*(M)$^+$ found
a	1-3	1053.14	1056.65
b	2-5, 4-5	1818.77	1827.58

Ac-WG<u>Pen</u>GGR ↓ GG<u>Pen</u>R ↓ CGGR ↓ GG<u>Pen</u>GWR ↓ GG<u>Dtaa</u>GW-NH2

<u>1</u> <u>2</u> 3 <u>4</u> <u>5</u>

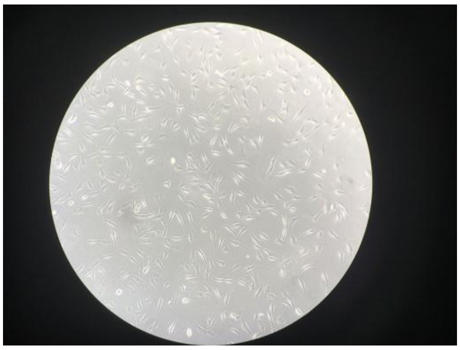

2a

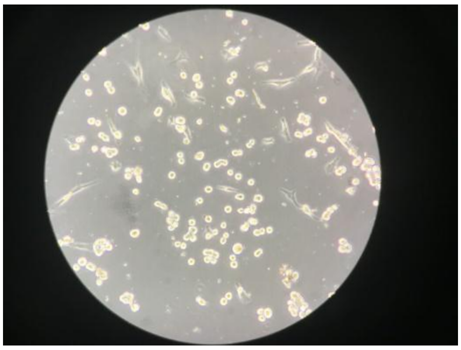

4a

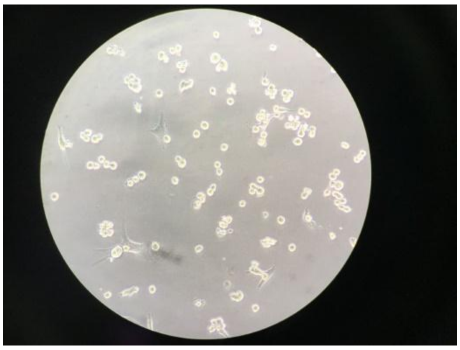

c(RGDyK)

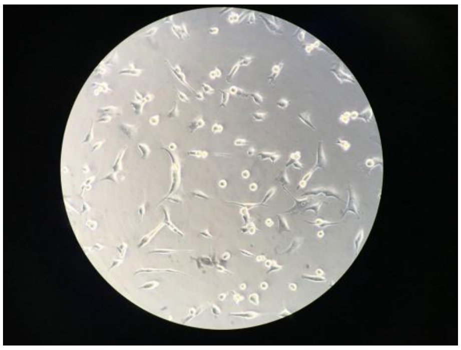

5a

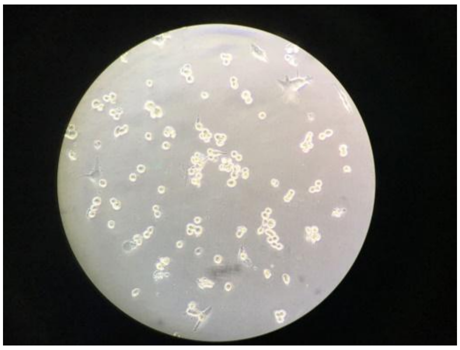

5b

Figure S18. The ability of 2a, 4a, c(RGDyK), 5a and 5b to block U87 cells adhesion were shown by inverted optical microscope.
